# Rab9 depletion enhances human adenovirus type 26 transduction efficiency through increased internalization and reduced late endosomal/lysosomal retention

**DOI:** 10.1371/journal.ppat.1014028

**Published:** 2026-08-10

**Authors:** Isabela Drašković, Davor Nestić, Lucija Lulić Horvat, Jelena Martinčić, Mario Stojanović, Gabriela N. Condezo, Klara Kašnar, Esther González-Almela, Darío Lago, Carmen San Martín, Jerome Custers, Dragomira Majhen

**Affiliations:** 1 Laboratory for Cell Biology and Signalling, Division of Molecular Biology, Ruđer Bošković Institute, Zagreb, Croatia; 2 Department of Macromolecular Structures, Centro Nacional de Biotecnología (CNB-CSIC), Madrid, Spain; 3 Janssen Vaccines and Prevention B.V., Leiden, The Netherlands; Brown University, UNITED STATES OF AMERICA

## Abstract

Understanding intracellular trafficking is central to decoding viral pathogenesis and engineering optimized viral vectors. How a virus or vector is routed through the endocytic pathway directly dictates its genome release, immune sensing, and overall transduction efficiency. Human adenovirus type 26 (HAdV-D26) presents a promising platform for vector design due to its low preexisting immunity, potent immune stimulation, scalable production, and versatile genetic engineering capacity. Although increasingly significant, the fundamental mechanisms governing HAdV-D26 intracellular trafficking are still not fully understood. Our study demonstrates that compared to well-described human adenovirus type 5 (HAdV-C5), HAdV-D26 undergoes prolonged intracellular trafficking, transiently localizing to early endosomes before residing in late endosomes/lysosomes for up to four hours post-infection. Inhibition of lysosomal acidification modestly enhances HAdV-D26 transduction efficiency, whereas blocking transport from early to late endosomes/lysosomes does not. Strikingly, Rab9 knockdown reduces HAdV-D26 late endosomal/lysosomal localization while increasing both virus internalization and genome delivery to the host cell nucleus. These findings indicate that late endosomal sorting pathways actively influence HAdV-D26 infection outcomes. By identifying a previously unappreciated role for Rab9 in adenovirus transduction, our results provide new mechanistic insight into HAdV-D26 intracellular trafficking, highlight serotype-specific differences in adenovirus entry pathways, and identify endosomal trafficking steps that may be targeted to improve adenoviral vector performance.

## Author summary

Human adenovirus type 26 is an important platform for vaccines and gene therapy due to its low preexisting immunity and versatility as a vector. Despite its growing importance, how HAdV-D26 enters and moves within host cells is still not well understood. In this study, we show that compared to HAdV-C5, HAdV-D26 exhibits prolonged intracellular trafficking, transiently passing through early endosomes and accumulating in late endosomes and lysosomes for several hours after entry. We further identify the host trafficking protein Rab9 as an important regulator of HAdV-D26 infection, as altering Rab9 levels changes viral internalization, late endosomal localization, and delivery of the viral genome. These findings reveal previously unrecognized mechanisms that control adenovirus intracellular trafficking and demonstrate that different adenovirus types use distinct intracellular trafficking routes. Understanding these pathways provides insights that may guide the development of more effective and better-controlled adenovirus-based vaccines and gene delivery vectors.

## Introduction

The benefits of human adenovirus (HAdV) vectors have been widely recognized in gene therapy, vaccination, and cancer treatment due to their high transduction efficiency, ability to infect both dividing and non-dividing cells, and capacity for large-scale production [1]. Traditionally, HAdV-C5 has been the vector of choice; however, recent research has increasingly focused on alternative adenovirus types. This shift is primarily driven by the prevalence of pre-existing immunity to HAdV-C5 in the general population [2], which can significantly diminish its therapeutic efficacy.

Human adenovirus type 26 is recognized as a promising vector for vaccination, largely due to its low seroprevalence and strong immunogenicity [3]. The versatility of HAdV-D26 in addressing global health emergencies was demonstrated by its regulatory approval as a vaccine vector for both COVID-19 (Ad26.COV2.S) and Ebola virus disease (Ad26.ZEBOV). Despite its growing importance, the fundamental mechanisms underlying HAdV-D26 intracellular trafficking remain incompletely understood. Recent studies indicate that the successful cell entry and transduction efficiency of HAdV-D26 in epithelial cells are strongly dependent on the expression of αvβ3 integrin, which facilitates virus internalization [4]. While αvβ3 integrin-mediated HAdV-D26 infection involves dynamin-2 and is caveolin-1-dependent, HAdV-D26 infection of epithelial cells with low expression of αvβ3 integrin involves dynamin-2 and clathrin, and is caveolin-1-independent. Downregulation of clathrin resulted in increased HAdV-D26 infection due to elevated expression of αvβ3 integrin, whereas inhibition of clathrin-coated pits disabled HAdV-D26 transport through the cytoplasm, indicating that in cells with low expression of αvβ3 integrin, HAdV-D26 infection may be clathrin-mediated [5]. Data regarding HAdV-D26 intracellular trafficking are limited, with only one study reporting that HAdV-D26 accumulates in the late endosomal compartment more extensively than HAdV-C5 at 2–8 h post-infection [6].

After internalization, adenoviruses reside within primary endocytic vesicles, endosomes, from which they need to escape to reach the nucleus. Early endosome escape in the case of HAdV-C5 is mediated by the viral membrane lytic protein VI [7], however, a discussion regarding the role of acidic pH in endosomal membrane penetration of HAdV-C2 and -C5 is still active [8]. Different adenovirus types exhibit different trafficking pathways within the host cell and reside within different endosomes. Species B types accumulate in lysosomes, whereas species C types traffic rapidly to the nuclear envelope, revealing different kinetics of endosomal escape. The optimum pH for membrane lysis matches that of early sorting endosomes in adenoviruses of species C, and that of late endosomes or lysosomes for species B [9].

Endosomes continuously remodel and mature by lowering the pH inside their lumen, moving toward the perinuclear region, and altering their shape. Several proteins are dynamically exchanged on the endosomal membrane, notably members of the Rab family of small GTPases. The human genome encodes over 60 distinct Rab proteins, each playing specialized roles in membrane trafficking. Among these, Rab5 is crucial for the biogenesis and function of early endosomes, orchestrating their formation and maturation, while Rab7 and Rab9 are primarily involved in regulating the activities of late endosomes, ensuring proper cargo sorting and transport within the endocytic pathway [10]. In the context of adenovirus cell entry, the role of Rab5 and Rab7 has been reported in the regulation of adenovirus endocytosis. Specifically, Rab5 was shown to control the endocytosis and trafficking of the escape-defective, temperature-sensitive HAdV-C2_ts1 (Ad2-ts1) mutant to late endosomes, but did not influence the infection process of wild-type HAdV-C2. On the other hand, sorting of HAdV-C2_ts1 to late endosomes was independent of Rab7, while HAdV-C2 and -C5 infection was independent of EEA1, a marker of early endosomes [11]. While HAdV-C2_ts1 is degraded in lysosomes [12], HAdV-B7 retention in a late endosomal compartment did not cause a loss in infection efficiency. Tracking the trafficking of HAdV-B7 to late endosomes revealed partial co-localization with late endosomal and lysosomal marker proteins, including Rab7, mannose-6-phosphate receptor, and LAMP1 [13]. So far, no studies have reported implication of Rab9 in the adenovirus infection pathway.

Understanding adenovirus intracellular trafficking is essential because efficient therapeutic gene delivery depends on successful cellular entry, endosomal escape, and nuclear import. HAdV-D26 intracellular transport mechanisms, particularly the functional role of endosomes as well as the role of endolysosomal regulators like Rab GTPases, remain poorly characterized. In this work, we describe the intracellular localization of HAdV-D26 and determine which specific endosomal compartments HAdV-D26 associates with during its intracellular trafficking. We also investigate the role of endosomes in HAdV-D26 transduction efficiency. Our study reveals that, compared to HAdV-C5, HAdV-D26 exhibits prolonged intracellular trafficking characterized by transient early endosome localization and extended lysosomal residence, resulting in a trend toward decreased nuclear genome delivery. We demonstrate that Rab9 knockdown enhances HAdV-D26 transduction efficiency by increasing virus internalization and nuclear delivery while reducing lysosomal retention. These findings reveal a previously unrecognized role for the Rab9-mediated pathway in HAdV-D26 trafficking and provide mechanistic insights to optimize adenoviral vectors for therapeutic applications.

## Results

### HAdV-D26 displays prolonged intracellular trafficking, resides in different cellular compartments during the first hour of internalization, and exhibits aberrant nuclear DNA delivery compared to HAdV-C5 in epithelial cells

**Fig 1:**
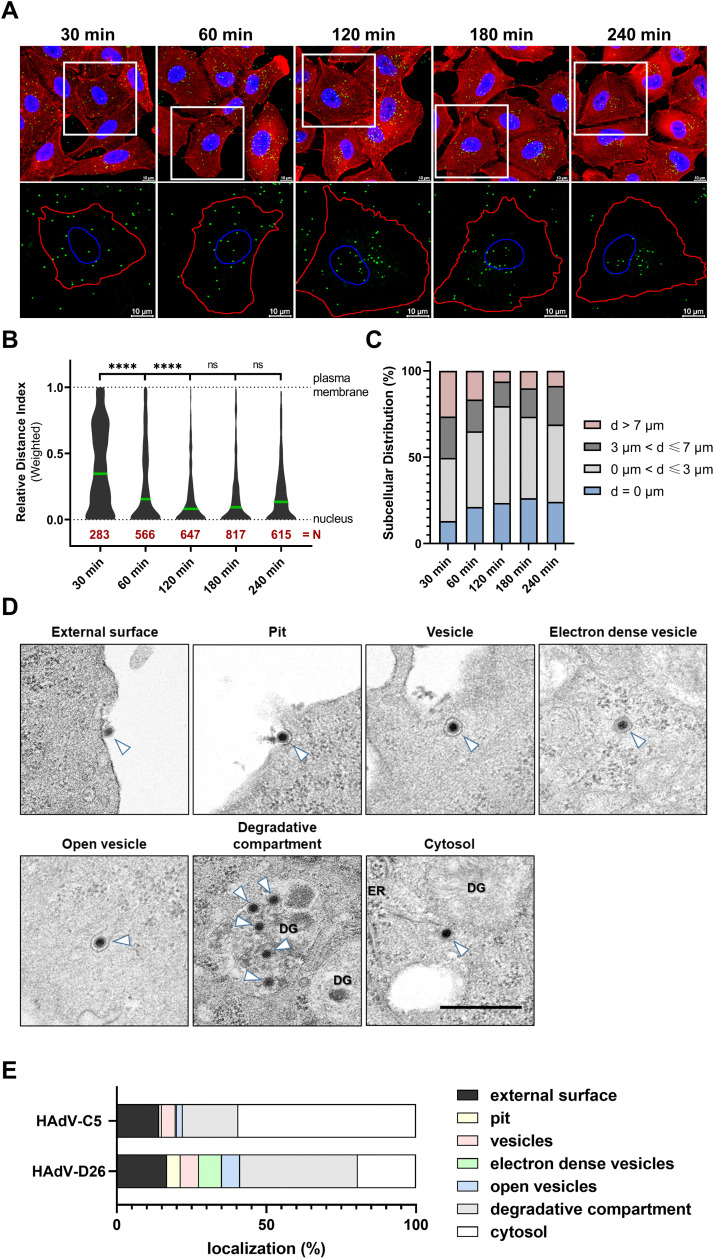
Intracellular trafficking of HAdV-D26 in epithelial cells. (A) Intracellular trafficking of HAdV-D26 in A549 cells observed by confocal microscopy. Cells were incubated on ice with fluorescently labeled HAdV-D26 (green; MOI 10^5^ vp/cell) for 45 min, then 10 min at 37°C, followed by washing with fresh medium to remove unbound viruses. After additional incubation at 37°C for the indicated times, cells were fixed and stained with phalloidin-AF555 (red) and DAPI (blue). Representative confocal images are shown (scale bar = 10 μm). The lower panels show areas selected from the upper panels at larger magnification, with marked edges of the nucleus (blue, based on DAPI) and cell (red, based on phalloidin). (B) Kinetics of HAdV-D26 intracellular trafficking shown in (A). Intracellular localization of individual virions was quantified using the Relative Distance Index (Weighted). An index value of 0 represents particle position at the nucleus, while a value of 1 represents the plasma membrane. Green line represents median. The exponential weighting was applied to compensate for cellular asymmetry. (C) Subcellular distribution of HAdV-D26 shown in (A). Bars represent the percentage of virions (N = 283-817 per time point) localized within four mathematically defined zones based on their absolute distance (d) from the nucleus: nuclear fraction (d = 0 µm), perinuclear region (0 < d ≤ 3 µm), mid-cytoplasm (3 < d ≤ 7 µm) and the cortical periphery (d > 7 µm). (D) HAdV-D26 resides in different cellular compartments during the first hour of internalization and intracellular trafficking. Representative TEM images of intracellular trafficking of HAdV-D26 in A549 cells. Cells were incubated on ice with HAdV-D26 (MOI 2x10^5^ vp/cell) for 45 min, then 10 min at 37°C, followed by washing with fresh medium to remove unbound virus particles, and then incubated for 1 h at 37°C. Cells were then fixed and prepared for TEM. Scale bar = 500 nm. Endoplasmic reticulum (ER); Degradative Compartment (DG). White arrowheads denote virus particle. (E) Quantification of HAdV-D26 and HAdV-C5 localization in different cellular compartments in A549 cells shown in (D) and (S1D Fig). Number of cells for HAdV-D26: 56; number of viral particles for HAdV-D26: 262 total viral particles. Number of cells for HAdV-C5 42; number of viral particles for HAdV-C5: 503 total viral particles.

The only study to date addressing the intracellular trafficking of HAdV-D26, indicates that this virus accumulates in the late endosomal compartment [6]. To investigate this behavior in more detail we performed confocal microscopy and transmission electron microscopy (TEM) analysis of HAdV-D26 intracellular trafficking in A549 cells. In contrast to well-characterized intracellular trafficking of HAdV-C5, which typically reaches the nucleus within approximately 60 min *in vitro* (S1A–S1C Fig), most HAdV-D26 particles remained dispersed throughout the cytoplasm 60 min after transduction. Even at 240 min p.i. (post-infection), there is no significant accumulation of HAdV-D26 at the nucleus, and most viral particles appear grouped on one side in the wider perinuclear area (Fig 1A–1C). To determine whether HAdV-D26 is free in the cytosol or associated with membrane-bound organelles, we investigated its intracellular trafficking in A549 cells 60 min after internalization using TEM. HAdV-D26 was observed at the plasma membrane, in several distinct cellular compartments including endosomes and degradative compartments, as well as free in the cytosol (Fig 1D). Quantitative analysis of viral localization revealed that the majority of the HAdV-D26 particles were found at the cell membrane, within membranous compartments and in the cytosol, with nearly 40% localized in the degradative compartment. ‘Degradative compartment’ is here used as a collective term that could include autolysosomes, late endosomes, lysosomes, amphisomes, and degradative autophagic vacuoles [14]. At the same time more than 60% of HAdV-C5 is found free in the cytosol (Figs 1E and S1D). These results suggest that after internalization, a distinct intracellular trafficking pattern of HAdV-D26 in A549 cells may contribute to its lower transduction efficiency compared to HAdV-C5 [4].

**Fig 2:**
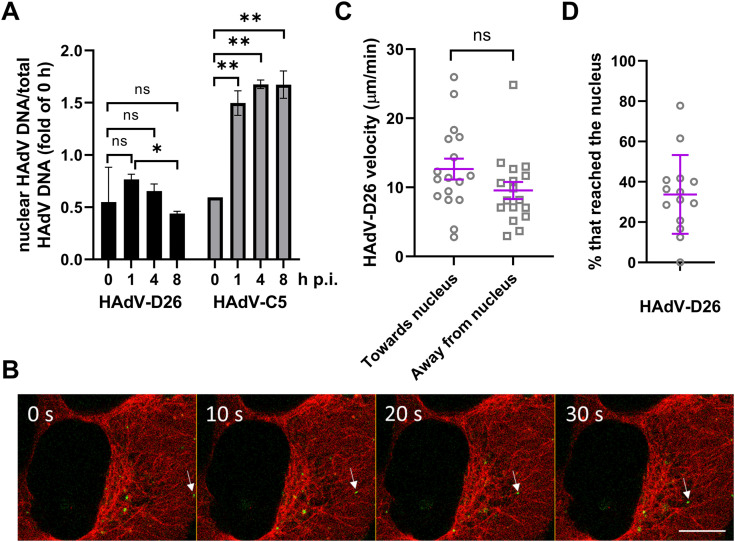
HAdV-D26 shows impaired nuclear entry dynamics. A) HAdV-D26 genome delivery into the host nucleus. A549 cells were infected with HAdV-C5 or HAdV-D26, MOI 10^4^ vp/cell for 45 min on ice to ensure uniform virus entry into the cell. Five min after incubation at 37˚C unbound viruses were removed and fresh medium was added, followed by incubation at 37°C. Cells were harvested at defined time intervals. DNA was extracted from the total cells or isolated nuclei. Viral and cellular DNA were quantified by qPCR using primers for the CMV promoter present in the viral DNA or the cellular GAPDH gene. Results are presented as a ratio of viral DNA measured in the nuclear fraction to the total fraction, normalized to the value for 0 h. Data are presented as mean values ± SD from three experiments. B) HAdV-D26 spatial distribution in U2OS mCherry-α-tubulin cells. U2OS cell with stable expression of mCherry-α-tubulin (red) infected with fluorescently labeled HAdV-D26 (green), MOI 5x10^4^ vp/cell, representing fast movement of HAdV-D26 (white arrow) towards the nucleus. Scale bar represents 10 µm. C) Average velocity of rapid HAdV-D26 movements directed towards or away from the nucleus in U2OS cells (p = 0.12, unpaired t- test, 13 cells, 17 viral particles in both directions). D) Percentage of HAdV-D26 viral particles that reached nuclear surface of U2OS cells within an hour after infection (total of 206 viral particles from 14 cells). **, P < 0.01.

To gain better insight into the kinetics of genome delivery by HAdV-D26, we quantified its nuclear entry in A549 cells using qPCR with primers specific for the CMV promoter present in the viral genome. HAdV-D26 DNA was quantified at 0 h, 1 h, 4 h, and 8 h p.i. from both total cell lysate and isolated nuclei. HAdV-D26 genome delivery to nuclei was calculated as the ratio of HAdV-D26 DNA in a nuclear fraction to HAdV-D26 DNA in total cell lysate. Most of the HAdV-D26 DNA was delivered to the nucleus within 1 h (Fig 2A); however, at later time points, we observed a trend toward decreased nuclear delivery of HAdV-D26 DNA, which may reflect DNA degradation or impaired nuclear import. These data are in accordance with the modest transduction efficiency of HAdV-D26 compared to HAdV-C5 and -B35 [4]. Contrary to HAdV-D26, HAdV-C5 genome delivery to the nucleus slightly increased over time (Fig 2A).

After endosomal escape adenoviruses use motor proteins for bidirectional trafficking on microtubules. In non-polarized cells, dynein-based minus-end directed transport prevails over the plus-end directed kinesin‐based transport, leading to the virus enrichment near the centrosome proximal to the nucleus [15]. Therefore, we tracked the movements of internalized fluorescently labeled HAdV-D26 and measured the velocity of its directed motions towards or away from the nuclear surface. For this assay we used U2OS cell line stably expressing mCherry-α-tubulin as a complementary model. The HAdV-D26 transduction efficiency in these cells was comparable to that in A549 (S2 Fig). HAdV-D26 virions usually followed the path of microtubule fibers (Fig 2B) and average HAdV-D26 velocity was 12 ± 6 µm/min towards the nucleus and 9 ± 5 µm/min away from the nucleus (Fig 2C). Observed velocities are comparable to those we measured for HAdV-C5 in the same model (12 ± 5 µm/min towards the nucleus and 13 ± 3 µm/min away from the nucleus) (S3 Fig). Although HAdV-D26 exhibits movements towards the nucleus, only an average of 34% viral particles among those with rapid movements reached the nuclear surface within one hour from infection (Fig 2D).

### HAdV-D26 transiently visits the early endosome and resides in lysosomes up to 240 min post-infection

**Fig 3:**
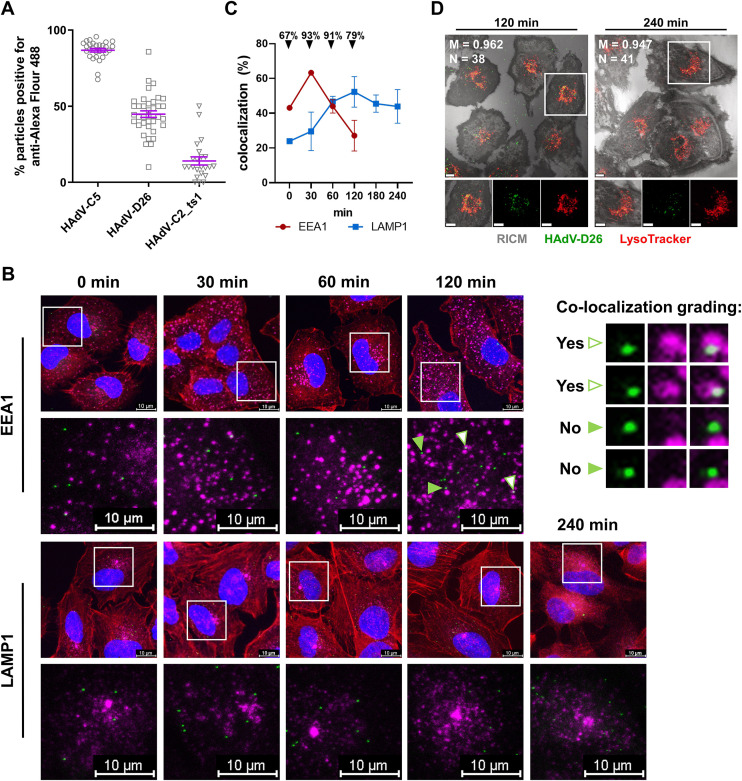
HAdV-D26 shows localization within lysosomes as long as 240 min p.i. A) Membrane penetration of HAdV-C5, HAdV-D26 and HAdV-C2_ts1 in A549 cells measured by SLO penetration assay 45 min p.i. Quantification of cytosolic virus particles in cells infected with Alexa Fluor 488-labeled HAdVs. Intact cells were treated with SLO and then incubated with anti-Alexa Fluor 488 antibody which was detected by secondary Alexa Fluor 594-conjugated antibody. The plot shows the percentages of virus particles positive for anti-Alexa Fluor 488 antibody. One dot represents one cell. Data are presented as mean values ± SEM. B) Localization of HAdV-D26 within early and late endosomes/lysosomes upon internalization. Representative confocal images are shown. Cells were incubated on ice with fluorescently labeled HAdV-D26 (green; MOI 10^5^ vp/cell) for 45 min, then 10 min at 37°C, followed by washing with fresh medium to remove unbound viruses. After additional incubation at 37°C for the indicated times, cells were fixed and stained with an antibody against EEA1 or LAMP1 (magenta), phalloidin-AF555 (red), and DAPI (blue). The upper right panel shows examples of co-localization grading: white arrows indicate co-localization, whereas green arrows indicate lack of co-localization. C) Quantification of co-localization of HAdV-D26 with either EEA1 or LAMP1 at different time points shown in (B). Percentages in black above the plot show the total amount of viruses found in any endosome (either EEA1 or LAMP1 positive). Data are presented as mean values ± SD from two independent experiments. The cell number that was analyzed is as follows: 1) for EEA1: 21 cells at 0 min, 78 cells at 30 min, 46 cells at 60 min, 65 cells at 120 min; 2) for LAMP1: 43 cells at 0 min, 78 cells at 30 min, 50 cells at 60 min, 90 cells at 120 min. D) Co-localization of HAdV-D26 with LysoTracker in live A549 cells. Cells were incubated on ice with fluorescently labeled HAdV-D26 (green) for 45 min, then 10 min at 37°C, followed by washing with fresh medium to remove unbound viruses, and then incubated at 37°C for the indicated times. In the last 30 min of incubation, Lysotracker Deep Red solution was added to the medium. After incubation, cells were washed with fresh medium and immediately observed by a confocal microscope. Reflection Interference Contrast Microscopy (RICM) is shown in grey. Representative confocal images are shown (scale bar = 10 μm). N denotes number of analyzed cells, M denoted Manders’ coefficient.

It is known from the literature that HAdV-C2 and -C5 visit early endosomes but are not transported to late endosomes [11]. In order to identify cytosolic HAdV-D26 particles and distinguish them from those inside membranous compartments, we carried out streptolysin O (SLO) assay. SLO-mediated perforation of the plasma membrane is used to introduce antibodies into the cytosol, and accessibility of Alexa Fluor 488-labeled virus to anti-Alexa Fluor 488 antibodies distinguishes cytosolic from endosomal viruses in the permeabilized cells [16]. At 45 min p.i. only 44% of HAdV-D26 particles were positive for anti-Alexa Fluor 488 antibody representing cytosolic particles, an intermediate value between those of HAdV-C5 and the entry-defective HAdV-C2_ts1 (Figs 3A and S4). These data allowed us to conclude that the majority of HAdV-D26 particles linger within some endosomal compartment, however at this point we did not know which one. Thus, we revisited HAdV-D26 localization within early and late endosomes. For that purpose, we observed co-localization of HAdV-D26 and EEA1, a marker of early endosomes, during 120 min p.i., as well as co-localization of HAdV-D26 and LAMP1, a marker of late endosomes and lysosomes, up to 240 min p.i. (Fig 3B). Our confocal microscopy data indicated that 30 min p.i. about 63% of the incoming HAdV-D26 co-localized with EEA1 (Fig 3C). At later time points, co-localization with EEA1 decreased. However, even at 120 min p.i. about 27% of HAdV-D26 was still localized in early endosomes. At 30 min p.i. about 30% of HAdV-D26 co-localizes with LAPM1, indicating that already at early points of infection HAdV-D26 is found in late endosomes or lysosomes. At 120 min p.i., nearly 80% of HAdV-D26 remains within early or late endosomes/lysosomes, and even at 240 min p.i., over 50% still co-localizes with the late endosomal/lysosomal marker LAMP1 (Fig 3C). Retention of HAdV-D26 in late endosomes/lysosomes is in line with our TEM results where we observed HAdV-D26 in degradative compartment.

Since our TEM results showed significant accumulation of HAdV-D26 particles in structures consistent with degradative compartments, and because we saw co-localization of HAdV-D26 with LAMP1-positive endosomes, we wanted to further identify the compartment where HAdV-D26 is retained. Thus, we performed co-localization of HAdV-D26 with LysoTracker, a marker for lysosomes, using live confocal microscopy. A high degree of co-localization between HAdV-D26 and LysoTracker was observed at 120 and 240 min p.i. (Fig 3D), with mean Manders’ split coefficients (M1) of 0.962 ± 0.004 and 0.947 ± 0.008 respectively. These results further corroborate our data, indicating that HAdV-D26 resides within lysosomes for up to 240 min p.i., which might influence HAdV-D26 transduction efficiency.

### Inhibiting lysosomal acidification, but not transport from early to late endosomes/lysosomes, modestly increases HAdV-D26 transduction efficiency

**Fig 4:**
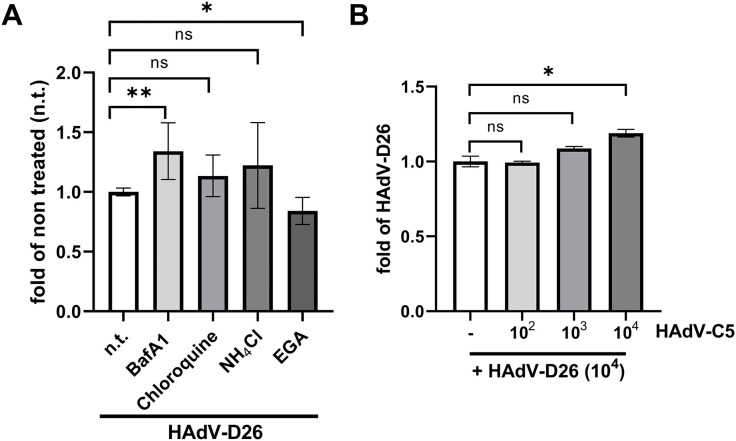
HAdV-D26 transduction efficiency is unaffected by endosomal pH modulation, inhibition of endosomal maturation, or co-infection with HAdV-C5. A) Increasing intralysosomal pH or inhibiting early-to-late endosome transition did not markedly affect HAdV-D26 transduction efficiency. Cells were pretreated with inhibitors (30 min, 37˚C) - bafilomycin A1 (10 nM), chloroquine (50 µM), NH_4_Cl (5 mM) or EGA (15 µM) and subsequently incubated with HAdV-D26 for one hour (MOI 10^4^ vp/cell) in medium with the specific inhibitor, after which the medium was changed and fresh medium was added. After 24 h of incubation, the fluorescence intensity of eGFP encoded by HAdV-D26 and the fluorescence intensity of cellular DNA labeled by Hoechst was measured. The results were calculated as the ratio of eGFP/Hoechst values and normalized to infected cells that were not treated (n.t.). B) Co-infection with HAdV-C5 does not influence HAdV-D26 transduction efficiency. Cells were infected either with HAdV-D26 (MOI 10^4^ vp/cell) alone or with a mixture of HAdV-D26 (MOI 10^4^ vp/cell) and HAdV-C5 at different MOIs (10^2^, 10^3^, 10^4^ vp/cell) (1 h, 37˚C), and then the medium was changed, and fresh medium was added. After 24 h of incubation, the fluorescence intensity of eGFP encoded by HAdV-D26 and the fluorescence intensity of cellular DNA labeled by Hoechst was measured. The results are presented as the ratio of eGFP/Hoechst values and normalized to cells infected with HAdV-D26. Data are presented as mean ± SD from two or three independent experiments in duplicates. *, P < 0.05; **, P < 0.01.

HAdV-D26 initially accumulates in early endosomes within 30 min p.i., then gradually shifts to late endosomes/lysosomes, where around 50% of the particles remain even at 240 min p.i. Since cargo directed to the lysosomes is typically destined for degradation, one can assume that prolonged residence of HAdV-D26 in late endosomes/lysosomes may lead to its degradation and may partly explain its low infectivity. To investigate whether disrupting lysosomal function affects HAdV-D26 infectivity, we measured its transduction efficiency in cells treated with the lysosomotropic agents such as bafilomycin A1 (BafA1), chloroquine, and ammonium chloride (NH_4_Cl). These inhibitors elevate intralysosomal pH, thereby impairing lysosomal function and disrupting autophagic protein degradation [17]. Treatment with all three inhibitors modestly increased transduction efficiency of HAdV-D26, where only treatment with bafilomicin A1 was statistically significant (Fig 4A). Nevertheless, the obtained data imply that at least some HAdV-D26 particles trafficking to late endosomes/lysosomes cannot complete the infection cycle.

We also investigated whether HAdV-D26 transport from early endosomes to late endosomes/ lysosomes would affect its transduction efficiency. For that purpose, we used EGA, an inhibitor of endosomal trafficking that specifically blocks transport to LAMP1-positive compartments, thereby impacting lysosomal function without disrupting other recycling pathways [18]. The late endosome trafficking inhibitor EGA only slightly decreased transduction efficiency with HAdV-D26 (Fig 4A), further corroborating our assumption that for efficient transduction, HAdV-D26 escapes from endosomes before reaching the lysosomes. Overall, these results indicate that trafficking from the early endosome to the lysosome is not essential for efficient HAdV-D26 transduction.

So far, our data suggest that HAdV-D26 might behave similarly to HAdV-C2_ts1, which does not efficiently escape from endosomes [19]. On the other hand, HAdV-C2 is liberated from the early endosome rather quickly and very efficiently. When HAdV-C2_ts1 was co-infected with HAdV-C2 its normally increased co-localization in early endosomes was significantly reduced [11], implying that HAdV-C2 facilitates the release of HAdV-C2_ts1 from early endosomes. Based on a similar concept, we performed a co-infection experiment using HAdV-D26 (encoding an eGFP reporter gene) and HAdV-C5 (encoding a β-galactosidase reporter gene) at increasing multiplicities of infection (MOI), and assessed HAdV-D26 transduction efficiency by quantifying eGFP expression. Co-infection of HAdV-D26 with HAdV-C5 at a high MOI of 10^4^ increased HAdV-D26 transduction efficiency by only 18%, while co-infection at lower MOIs had no significant effect on HAdV-D26 transduction efficiency (Fig 4B). These data indicate that HAdV-C5 endosome penetration does not liberate HAdV-D26 competent for productive transduction or that HAdV-D26 particles do not traffic through the same endosomal compartments as HAdV-C5.

### Functional Rab5, Rab7, and Rab9 are involved in HAdV-D26 transduction

**Fig 5:**
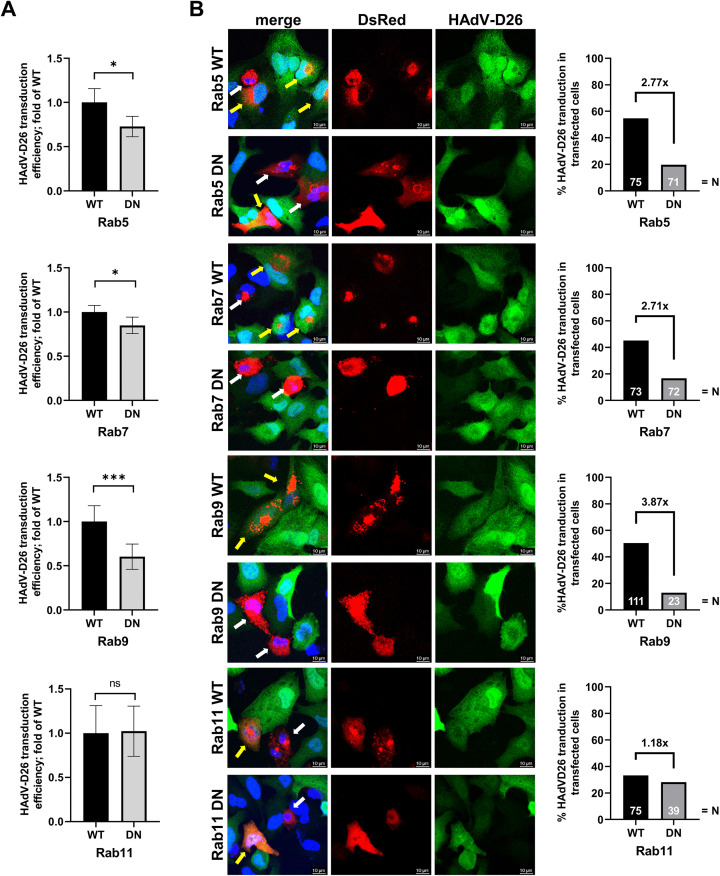
HAdV-D26 transduction efficiency partially depends on functional Rab5, Rab7 and Rab9 proteins. A549 cells were transfected with WT or DN Rab5, Rab7, Rab9 or Rab11 plasmids (48 h, 37°C), and subsequently incubated with HAdV-D26 for one hour, after which the medium was changed, and fresh medium was added. After 24 h, transduction efficiency was assessed by flow cytometry or confocal microscopy. A) HAdV-D26 transduction efficiency corresponding to the geometric mean of the fluorescence intensity of eGFP signal encoded by HAdV-D26 (MOI 10^4^ viral particles per cell) shown as a value relative to the corresponding WT sample. Data are presented as mean ± SD from three independent experiments in duplicates. B) Panels show representative cells from one experiment, where cells expressing a certain Rab protein (DsRed positive cells; red) were scored for HAdV-D26 infection (eGFP positive cells; green). Cell nuclei are shown in blue (DAPI). Yellow arrows indicate cells that are red/green positive (transfected and transduced cells), while white arrows indicate cells that are red positive (transfected cells) and green negative (non-infected cells). Scale bar, 10 μm. HAdV-D26 transduction efficiency (MOI 10^5^ vp/cell) is quantified as a percentage of the eGFP expressing cells transfected with Rab5, Rab7, Rab9 or Rab11 DN compared to eGFP expressing cells transfected with WT plasmids (right hand graphs). N represents the number of analyzed cells. *, P < 0.05; ***, P < 0.001.

After endocytosis, cargos are sorted into endosomes and directed to various destinations. Rab GTPases, localized to distinct endosomes, regulate these processes by controlling membrane budding, vesicle formation, motility, tethering, and fusion through effector recruitment [10]. It has been shown that HAdV-C2 and -C5 reach the cytosol in a Rab5-independent manner, while HAdV-C2_ts1 depends on Rab5 for endocytosis and transport to late endosomes and lysosomes. At the same time, sorting of HAdV-C2_ts1 to late endosomes was independent of Rab7 [11]. To test if functional Rab proteins are involved in HAdV-D26 intracellular trafficking, we determined HAdV-D26 transduction efficiency in cells transfected with wild-type (WT) or dominant-negative (DN) form of Rab5, Rab7, Rab9, or Rab11. Dominant-negative mutants work in cells by competing with endogenous Rabs for binding to GEFs (Guanine Nucleotide Exchange Factor) and since they cannot interact with downstream target proteins, they form ‘dead-end’ complexes further preventing the activation of endogenous Rabs [20]. The localization of transfected WT or DN forms and their transfection efficiencies are shown on S5 and S6 Figs respectively; note that WT proteins retain a punctate endosomal localization closely mimicking the physiological staining pattern of endogenous Rabs, whereas DN mutants display an altered, more diffuse distribution as previously reported [21]. Transduction efficiency of HAdV-D26 in cells transfected with DN form of Rab5, Rab7, or Rab9 measured by flow cytometry (Fig 5A) and confocal microscopy (Fig 5B) was partially decreased, while the Rab11 DN did not influence HAdV-D26 transduction efficiency. The most pronounced effect was observed in cells expressing Rab9 DN, where HAdV-D26 transduction efficiency was decreased by approximately 40% compared to cells transfected with Rab9 WT. These results suggest that HAdV-D26 transduction efficiency partially depends on functional Rab5, Rab7, and Rab9 proteins.

### Rab9 knockdown decreases HAdV-D26 localization in the lysosome

**Fig 6:**
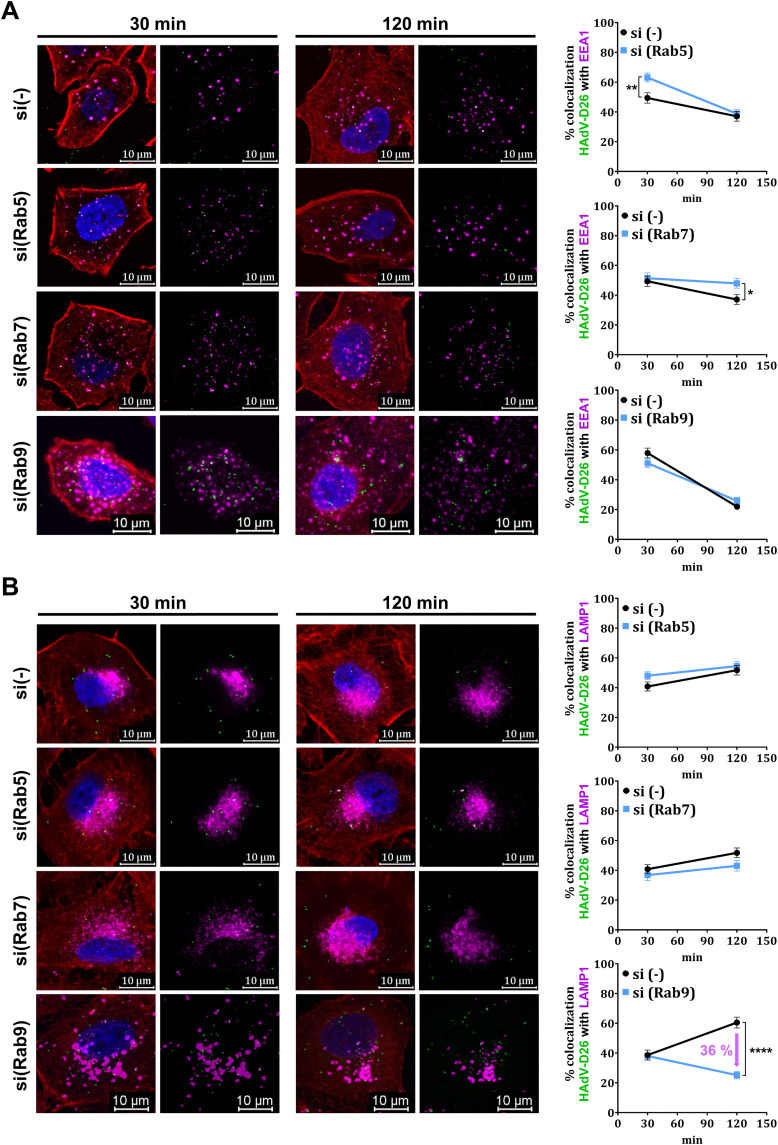
Rab5 and Rab7 knockdown increase HAdV-D26 localization within early endosomes, while Rab9 knockdown decreases HAdV-D26 localization within late endosomes/lysosomes. Cells were transfected with specific siRNA at the final concentration of 10 nM for Rab5 and Rab7, and 5 nM for Rab9, and 48 h later incubated with fluorescently labeled HAdV-D26 (green; MOI of 10^5^ vp/cell) for 45 min on ice to ensure uniform virus entry into the cell and subsequently transferred to 37˚C for 10 min. Unbound viruses were washed, fresh medium was added, and cells were returned to 37˚C for 30 min or 120 min, after which they were fixed and stained with antibody against (A) EEA1 (magenta) or (B) LAMP1 (magenta). F-actin is shown in red, and the nucleus in blue. Representative confocal images are shown. Scale bar = 10 μm. Rab5 and Rab7 knockdown experiments were performed in parallel using a shared si(-) control, whereas Rab9 experiments were conducted independently with a batch-matched si(-) control. Graphs on the right show quantification of HAdV-D26 co-localization with EEA1 or LAMP1 at 30 and 120 min following knockdown of the indicated Rab protein. Co-localization was quantified by counting the number of virus particles overlapping with EEA1- or LAMP1-positive structures per cell. Data represent the mean ± SEM from at least 30 cells analyzed per condition. *, P < 0.05; **, P < 0.01; ****, P < 0.0001.

To investigate further the role of Rab proteins in HAdV-D26 intracellular trafficking, we analyzed HAdV-D26 co-localization with EEA1 in cells transfected with siRNA specific for Rab5, Rab7, or Rab9 (Rab5, Rab7 or Rab9 knockdown efficiency is shown in S7 Fig). The results (Fig 6A) show that knockdown of Rab5 protein in A549 cells increases the localization of HAdV-D26 in the early endosome. Namely, at 30 min p.i., 13% more HAdV-D26 localized to EEA1-positive endosomes in Rab5-knockdown cells compared to those transfected with scrambled siRNA. However, by 120 min p.i., HAdV-D26 localization in EEA1-positive endosomes was similar between Rab5-knockdown and control cells (si(-)). Rab7 knockdown in A549 cells did not affect HAdV-D26 localization in early endosomes at 30 min p.i., but during the following 90 min, it increased retention of HAdV-D26 in early endosomes compared to control cells.

At specific time points, increased co-localization of HAdV-D26 with EEA1 in Rab5 or Rab7 knockdown cells suggests that, under normal conditions, HAdV-D26 might be liberated from Rab5 and Rab7 positive early endosomes. Knockdown of Rab9 does not change the percentage of HAdV-D26 co-localization with EEA1 marker, indicating that Rab9 does not interfere with HAdV-D26 trafficking within early endosomes.

Next, we analyzed HAdV-D26 co-localization with LAMP1 in cells transfected with siRNA specific for Rab5, Rab7, or Rab9 (Fig 6B). Knockdown of Rab5 protein in A549 cells modestly increased the localization of HAdV-D26 to late endosome/lysosome at 30 min p.i., with 10% more virus localized in LAMP1-positive endosomes compared to cells transfected with scrambled siRNA. At 120 min p.i., HAdV-D26 localization in LAMP1-positive endosomes was similar between Rab5-knockdown and control cells. In contrast, Rab7 knockdown in A549 cells reduced the proportion of HAdV-D26 in late endosomes/lysosomes at 120 min p.i. from 52% to 42%. This decrease in localization in LAMP1 positive endosomes is even more obvious in cells with Rab9 knockdown, where localization of HAdV-D26 120 min p.i. decreased from 60% to 24%. Decreased co-localization of HAdV-D26 with LAMP1 in Rab7- or Rab9-knockdown cells suggests that Rab7 and Rab9 might promote HAdV-D26 delivery to late endosome/lysosome. Knockdown of Rab5 does not change the percentage of HAdV-D26 co-localization with LAMP1 marker, indicating that Rab5 does not interfere with HAdV-D26 trafficking within late endosomes/lysosomes.

### Rab9 knockdown increases HAdV-D26 transduction efficiency

**Fig 7:**
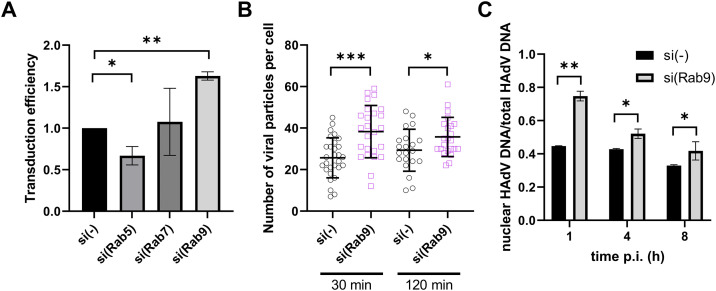
Knockdown of Rab9 increases internalization and transduction efficiency of HAdV-D26. A) Transduction efficiency of HAdV-D26 in A549 cells downregulated for Rab5, Rab7, or Rab9. Cells were transfected with specific siRNA at a final concentration of 10 nM for Rab5 and Rab7, and 5 nM for Rab9, and 48 h later infected with HAdV-D26 at an MOI of 10^4^ vp/cell. Transduction efficiency was measured as eGFP fluorescence 48 h p.i. The results are obtained by flow cytometry and presented as mean ± SD from three independent experiments normalized to control, i.e., cells transfected with scrambled siRNA. B) Internalization of HAdV-D26 in A549 cells downregulated for Rab9. Cells were transfected with specific Rab9 siRNA, 5 nM, and 48 h later infected with fluorescently labeled HAdV-D26, MOI 10^5^ vp/cell, for 45 min on ice to ensure uniform virus entry into the cell and subsequently transferred to 37˚C for 10 min. Unbound viruses were washed, fresh medium was added, and cells were returned to 37˚C for 30 min or 120 min, after which they were fixed and imaged by confocal microscopy. Quantification is presented as the number of viral particles per cell with mean ± SD. C) HAdV-D26 genome delivery into the host nucleus in Rab9 downregulated cells. Cells were transfected with scrambled siRNA or Rab9 siRNA at a final concentration of 5 nM, and 48 h later infected with HAdV-D26, MOI 10^4^ vp/cell for 45 min on ice to ensure uniform virus entry into the cell. Five min after incubation on 37˚C unbound viruses were removed, and fresh medium was added, followed by incubation at 37°C. Cells were harvested at defined time intervals. DNA was extracted from the total cells or isolated nuclei. Viral and cellular DNA were quantified by qPCR using primers for the CMV promoter present in the viral DNA or the cellular GAPDH gene. Results are presented as a ratio of viral DNA measured in the nuclear fraction to the total fraction, normalized to the corresponding si(-) value. Data are presented as mean values ± SD from three experiments. Subsequently, the same experimental setup was performed as described in Fig 2A. *, P < 0.05; **, P < 0.01, ***, P < 0.001.

Since we saw that functional Rab proteins are involved in HAdV-D26 transduction and that knockdown of Rab proteins influences HAdV-D26 transport through the endosomes, we wanted to investigate if depleting different Rab proteins would also play a role in the transduction efficiency of HAdV-D26. Thus, we downregulated Rab5, Rab7, or Rab9 by using specific siRNAs and measured the transduction efficiency of HAdV-D26. As shown in Fig 7A, a decrease of approximately 30% in HAdV-D26 transduction efficiency was observed upon Rab5 downregulation, while downregulation of Rab7 did not influence HAdV-D26 transduction efficiency. On the other hand, downregulating Rab9 increased HAdV-D26 transduction efficiency by 62%. Increased adenovirus transduction efficiency can be a consequence of increased internalization or increased adenovirus genome delivery to the host nucleus [22]. Therefore, we first investigated whether decreased expression of Rab9 influenced HAdV-D26 internalization. We quantified the number of internalized fluorescently labeled HAdV-D26 30 and 120 min p.i. and observed that downregulation of Rab9 increased HAdV-D26 internalization in average by more than 30% (Fig 7B). Downregulating Rab9 did not alter the expression levels of αvβ3 integrin, CAR or CD46 (S8 Fig). Knowing that Rab9 knockdown increases HAdV-D26 escape from lysosomes, we also wanted to investigate if Rab9 knockdown would change the amount of HAdV-D26 genome imported into the host nucleus. For that purpose, we measured HAdV-D26 genome delivery to the nucleus in A549 cells transfected with scrambled or Rab9 siRNA and observed an increase in HAdV-D26 DNA nuclear import in cells with Rab9 knockdown (Fig 7C). All these results indicate that Rab9 influences HAdV-D26 transduction efficiency on several levels including virus internalization and genome delivery.

## Discussion

First isolated in 1956 from a stool sample of an infant [23], HAdV-D26 is a prominent vaccine vector due to its low human seroprevalence compared to HAdV-C5 [3]. It utilizes various entry receptors based on cell type, but overall low binding affinities necessitate a higher MOI for effective transduction [4,24,25]. Although adenoviruses typically enter cells via clathrin-mediated endocytosis [26,27], HAdV-D26 switches between caveolin-mediated and clathrin-influenced pathways depending on αvβ3 integrin expression [5]. HAdV-D26 intracellular trafficking is still poorly understood, with limited data suggesting late endosomal involvement [6]. Here, we investigated the post-internalization trafficking of HAdV-D26 in A549 cells, focusing on the role of endosomal compartments in viral trafficking and transduction efficiency.

Under *in vitro* conditions, HAdV-C2 and HAdV-C5 reach the nucleus within 60 min p.i. [28]. In contrast, here we showed that HAdV-D26 reaches the nucleus considerably more slowly. Fluorescence imaging revealed that at 60 min p.i. HAdV-C5 localizes at the nucleus while HAdV-D26 remains dispersed throughout the cytoplasm, and even at 240 min p.i. shows no pronounced nuclear accumulation, with particles instead clustering asymmetrically in the perinuclear region. This observation reflects nuclear targeting of HAdV-C5 and perinuclear accumulation of HAdV-D26. A trafficking pattern similar to that of HAdV-D26 has been reported for HAdV-B7, and Ad5f7 virions indicating altered endosomal escape [29], which may likewise apply to HAdV-D26. When imaged by TEM during the first 60 min p.i. we detected HAdV-D26 at the plasma membrane, within vesicular structures, and free in the cytosol. Nearly 40% of viral particles accumulated in degradative compartments, likely lysosomes, indicating prolonged endosomal retention, contrary to HAdV-C5, which escapes endosomes as early as 20 min after uptake [16].

We examined HAdV-D26 endosomal trafficking in more detail by assessing co-localization with early (EEA1) and late endosome/lysosome (LAMP1) markers during 240 min p.i. Within the first 120 min, ~ 80% of HAdV-D26 particles remained in early or late endosomes/lysosomes, indicating limited endosomal escape. Although HAdV-D26 and HAdV-C5 both initially localize to early endosomes, co-infection experiments showed that they likely do not share the same endosomal pathway, as the presence of HAdV-C5 did not enhance HAdV-D26 transduction. However, lack of increased transduction could also result from the cytosolic release of hyper stable HAdV-D26 particles that have failed to release protein VI. Over 40% of HAdV-D26 persisted in LAMP1- and LysoTracker-positive compartments for up to 240 min, suggesting impaired endosomal escape or trafficking into lysosomal compartments. Our imaging data align with a previous study proposing that HAdV-D26 accumulates in late endosomes 2–8 h p.i. [6]. Although these authors showed co-localization of HAdV-D26 with EEA1 and LAMP1 in A549 cells, they observed a much lower co-localization percentage of HAdV-D26 than we did.

Prolonged lysosomal residence has been reported for HAdV-C2_ts1, HAdV-B7, and the chimeric Ad5/35L vector. For HAdV-C2_ts1 and Ad5/35L this results in less efficient gene transfer than HAdV-C5. Specifically, HAdV-C2_ts1 lacks the L3/p23 protease, preventing endosomal escape [11], while many Ad5/35L particles are recycled to the cell surface despite reaching the nucleus via late endosomal pathways. HAdV-B7 naturally traffics through lysosomes without losing its viral genome. Here we demonstrated that HAdV-D26 is rather inefficient in nuclear delivery of its genome. The highest amount of HAdV-D26 DNA imported into the host nucleus was observed 1 h post-infection, while at later infection time points a downward trend of the DNA delivery was observed, indicating an issue with nuclear import. Adenoviral DNA nuclear import requires capsid disassembly and interactions between viral and host factors [15,30,31]. Disruption of these interactions can block nuclear entry, leaving viral genomes in the cytoplasm or making them subject to degradation. Since we did not measure any decrease in the total amount of HAdV-D26 DNA that entered the cell over the 8-h time course, we conclude that HAdV-D26 DNA is not degraded but rather stays in the cytoplasm. We saw that only approximately 34% of rapidly moving HAdV-D26 particles reach the nucleus which could also account for the low nuclear import efficiency.

Our results indicate that approximately 20% of HAdV-D26 is liberated from any endosome, while more than 40% is retained in late endosome/lysosome. We hypothesize that the remaining 40% is sorted to an unidentified endosome from where it is either released or retained. Similarly, in A549 cells, unreleased HAdV-C2 bypasses typical endosomal maturation, instead entering an unidentified non-permissive compartment or recycling back to the plasma membrane [32]. Since Rab11 DN had no influence on HAdV-D26 transduction efficiency and HAdV-D26 did not exhibit significant movement away from the nucleus, we conclude that HAdV-D26 does not follow a recycling endosome pathway.

Since imaging data alone do not inform on the fate of viral particles within degradative compartments (degradation or *en route* to productive infection), we utilized functional inhibitors. Impairment of lysosomal acidification modestly increased HAdV-D26 transduction implying that HAdV-D26 entrapped in the lysosomes is lost in terms of transduction, presumably due to degradation. Conversely, lysosomal residence may benefit HAdV-D26 vectors by facilitating MHC II antigen processing [33] and innate immune sensing [34] creating a self-adjuvanting effect that enhances adaptive immunity.

Rab proteins define endosomal identity and regulate vesicle trafficking [35]. In A549 cells, a significant portion of HAdV-D26 traffics to late endosomes/lysosomes, a process independent of Rab5 and Rab7 but dependent on Rab9, as Rab9 knockdown reduced HAdV-D26 co-localization with LAMP1 by over 35%. Rab9 knockdown also increased HAdV-D26 transduction efficiency, internalization, and genome import into the host nucleus. Since Rab9 knockdown did not change cell surface expression of CAR, αvβ3 integrin or CD46 our data suggest that the observed increase in HAdV-D26 internalization is not driven by receptor abundance, but rather by altered trafficking kinetics or membrane dynamics.

Rab9 facilitates late endosome-to-trans-Golgi trafficking and lysosomal degradation of cargo [36], while Rab9 depletion alters endosome maturation by enlarging late endosomes and disrupting cargo retention [37]. Therefore, Rab9 knockdown could increase HAdV-D26 infection by impairing the Rab9-dependent lysosomal degradation pathway, reducing lysosomal delivery of HAdV-D26 particles, and enabling their accumulation in enlarged endosomes, promoting cytoplasmic escape. Notably, DN Rab9 and Rab9 knockdown had divergent effects due to their distinct mechanisms. While siRNA-mediated knockdown reduces Rab9 levels potentially accelerating HAdV-D26 endosomal escape or shifting it to alternative routes, DN Rab9 sequesters GEFs, causing broader disruption of endosomal maturation. These pleiotropic effects likely impair the membrane remodeling necessary for HAdV-D26 penetration, thereby reducing infection.

Rab9 has been shown to mediate hepatitis B virus degradation by trafficking envelope proteins to lysosomes via the NDP52 receptor. Consequently, Rab9 depletion increases cellular HBV DNA, suggesting that inhibiting this degradation pathway promotes HBV replication [38]. Conversely, Rab9 knockdown blocks HPV entry by disrupting retromer-mediated endosome-to-Golgi transport, causing viral accumulation in endosomes [39]. Interestingly, GDP-bound form of Rab9 actually promotes HPV entry, whereas GTP-bound Rab9a inhibits it, indicating that Rab9 GTPase nucleotide state has non-intuitive effects on infection and can behave non-canonically when it comes to viral cell entry. To the best of our knowledge, Rab9 has not been previously described as a limiting factor for adenovirus transduction efficiency.

From the literature we know that Rab9 can act as an oncogenic driver and therapeutic target in cancers such as breast cancer [40], melanoma [41], and gastric cancer [42] where its knockdown affects proliferation, migration, or invasion. In this context, our finding that Rab9 downregulation enhances HAdV-D26 transduction suggests that cellular Rab9 levels may influence susceptibility to infection. This raises the possibility that cells with inherently low Rab9 expression, or those in which Rab9 is reduced as a consequence of prior treatments, may be more permissive to HAdV-D26-based vectors. However, this remains speculative and would require further investigation.

Our data indicate that in cells with endogenous Rab9 expression HAdV-D26 is retained in late endosomal/lysosomal compartments, correlating with reduced transduction efficiency. Our interpretation is that virions retained in lysosomes are largely non-productive and therefore effectively lost for functional transduction. This interpretation is further supported by our experiments using lysosomotropic inhibitors, where interference with lysosomal function led to a modest increase in infectivity, consistent with the idea that lysosomal targeting represents a non-productive pathway for HAdV-D26. Providing more details regarding the possible degradation of HAdV-D26 within the lysosomes was beyond the scope of this study, however, our data raise the question of why HAdV-D26 would end up in the lysosomes in the first place. It is possible that HAdV-D26 can use a receptor which would sort it to the lysosomes, however none of the molecules proposed so far to be involved in HAdV-D26 cell entry actively direct their ligands to lysosomes. HAdV-D26 was originally isolated from stool, although it is not classified as a classical enteric adenovirus. Its retention within late endosomes may nevertheless indicate an increased tolerance for acidic intracellular environments, potentially reflecting structural features compatible with gastrointestinal exposure. If HAdV-26 is more stable than HAdV-C5 or has a protein VI release defect, it may have a slower or less efficient escape mechanism compared to HAdV-C5, making it more sensitive to the kinetics of endo-lysosomal maturation.

Ultimately, our results raise the question of whether HAdV-D26 post cell entry partitions into two distinct populations: a functional fraction that escapes early endosomes (similar to HAdV-C5) and a non-functional fraction that follows a degradative route (similar to HAdV-C2_ts1). We hypothesize that Rab9 knockdown prevents the diversion of viral particles into a non-productive pathway, thereby streamlining HAdV-D26 toward the nucleus. However, this still needs to be confirmed.

**Fig 8:**
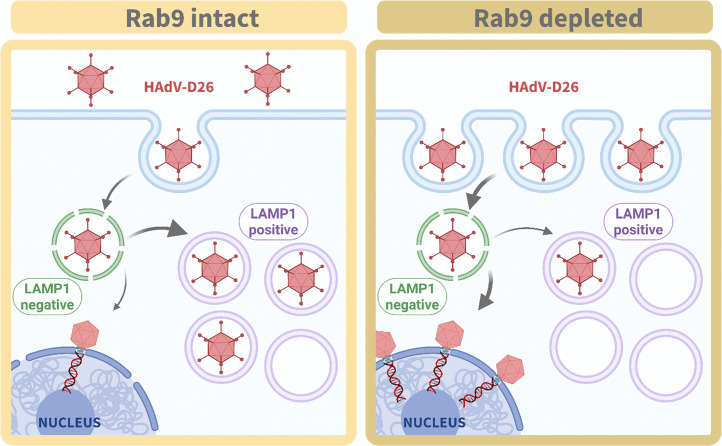
Schematic presentation of how Rab9 influences HAdV-D26 intracellular trafficking and transduction efficiency. Decreased expression of Rab9 allows for increased HAdV-D26 internalization but also increased liberation from currently unidentified endosomal compartment. Subsequently, HAdV-D26 retention in late endosome/lysosome (LAMP1-positive) is decreased, which could decrease potential HAdV-D26 degradation and promote better transduction efficiency. Created in BioRender. Nestic, D. (2026) https://BioRender.com/4ruxpso.

Our results extend current knowledge of adenovirus trafficking by providing new insights into post-entry HAdV-D26 dynamics and the role of Rab proteins in HAdV-D26 transduction efficiency. We highlight distinctive features of HAdV-D26, including Rab9-mediated prolonged lysosomal residence and limited genome delivery to the nucleus. Although Rab9 modulation affects viral internalization and transduction (Fig 8), the precise molecular mechanisms remain unclear. A limitation of our study is that the primary HAdV-D26 receptor is still unknown, thus epithelial cell models may not fully reflect *in vivo* conditions. Nevertheless, these findings offer valuable insights into virus-host interactions that can guide the design of improved adenoviral vectors.

## Materials and methods

### Cells, viruses, and antibodies

HEK293 (human embryonic kidney; ATCC CRL-1573) and A549 (human lung carcinoma; ATCC CCL-185) cells were obtained from the ATCC Cell Biology Collection and were cultured in antibiotic-free Dulbecco’s Modified Eagle’s Medium- high glucose (DMEM) supplemented with 10% (vol/vol) Fetal Bovine Serum (FBS) if not stated differently. Osteosarcoma U2OS cell line (female) stably expressing mCherry-α-tubulin was a kind gift from Marin Barišić (Danish Cancer Society Research Center, Copenhagen, Denmark) and Helder Maiato (Institute for Molecular Cell Biology, University of Porto, Portugal). U2OS cells were maintained in DMEM supplemented with 10% (vol/vol) FBS and 50 µg/mL geneticin. All cells were grown at 37°C in a humidified incubator with a 5% CO_2_ atmosphere. Replication-incompetent recombinant adenoviral vectors based on adenovirus types 2, 5 or 26 were previously constructed [43–45]. Viruses were propagated on HEK293 cells and purified by CsCl gradients. They carried an enhanced green fluorescent protein (eGFP) gene driven by the cytomegalovirus (CMV) promoter as a reporter gene. Fluorescent labeling of adenoviruses was previously described [4]. Primary antibodies used for immunofluorescence, co-localization analyses, western blot or flow cytometry were as follows: anti-EEA1 (#2411, Cell Signaling, SAD), anti-LAMP1 (ab24170, Abcam, UK), anti-Rab5 (#3547, Cell Signaling, SAD), anti-Rab7 (#9367, Cell Signaling, SAD), anti-Rab9A (#5118, Cell Signaling, SAD), anti-Alexa Fluor 488 recombinant F(ab)-rabbit monoclonal antibody (15H9L93, Invitrogen), anti-CAR (RcmB, Merck Millipore), anti-CD46 (MEM-258, Thermo Fisher Scientific) and anti-αvβ3 integrin (LM609, Merck Millipore).

### Transduction efficiency assay

Transduction efficiency was measured either by flow cytometry as previously described [5] or by spectrophotometer. For assaying by spectrophotometer, cells were seeded in 96-well plates at a density of 4x10^3^ cells per well, and 48 h later incubated with HAdV vectors at MOI of 10^4^ vp/cell. After 1 h at 37°C in a humidified incubator with a 5% CO_2_ atmosphere the medium was removed and replaced with of antibiotic-free DMEM supplemented with 10% (vol/vol) FBS and cultured for additional 24 h. Cells were then washed twice with PBS and eGFP expression was measured by spectrophotometer at 515 nm. Subsequently, after washing, cellular DNA was stained with Hoechst 33342 (5 µM) and the fluorescence intensity was measured at 455 nm. The results were calculated as the ratio of eGFP/Hoechst values.

For measuring transduction efficiency after interfering with endosome pH cells were pretreated with following inhibitors (30 min, 37˚C): bafilomycin A1 (10 nM), chloroquine (50 µM), NH_4_Cl (5 mM) or EGA (15 µM) and subsequently incubated with HAdV-D26 for one hour (MOI 10^4^ vp/cell) in medium with the specific inhibitor, after which the medium was changed and fresh medium was added. After 24 h of incubation, the fluorescence intensity of eGFP was measured and subsequently the fluorescence intensity of cellular DNA labeled by Hoechst.

For studying role of Rab proteins in HAdV-D26 transduction efficiency cells were transfected with DN or WT form of Rab5, Rab7, Rab9 or Rab11 purchased from Addgene: DsRed-Rab5 WT, #13050; DsRed-Rab5 DN, #13051; DsRed-Rab7 WT, #12661; DsRed-Rab7 DN, #12662; DsRed-Rab9 WT, #12677; DsRed-Rab9 DN, #12676; DsRed-Rab11 WT, #12679; DsRed-Rab11 DN, #12680. Plasmids were transfected into A549 cells using Lipofectamine (Thermo Fisher Scientific) according to the manufacturer’s protocol, and 48 h after cells were transduced with HAdV-D26.

Transfection with Rab WT or DN coding plasmids and transduction efficiencies after infection of transfected cells with HAdV-D26 were measured by flow cytometry using a FACSCalibur flow cytometer (BD Biosciences, USA). Data acquisition was performed using the BD CellQuest software package (BD Biosciences, USA). The number of acquired events per sample was 10,000. Data were analyzed using FCS Express 3 (De Novo Software, USA), and the gating strategy was established by first identifying the baseline cell population and eliminating cell debris using linear Forward Scatter (FSC) versus Side Scatter (SSC) dot plots. Within this viable cell gate, viral transduction efficiency was quantified based on the green fluorescence intensity of the eGFP signal encoded by the adenoviral vectors, recorded in the FL1 channel (excitation 488 nm, emission 530/30 nm filter). Results are expressed as relative transduction efficiency, calculated from the geometric mean of fluorescence intensity (MFI) of the eGFP signal and normalized to the corresponding control sample. To evaluate transfection efficiencies, the expression of DsRed-tagged Rab constructs (WT and DN) was analyzed in the FL2 channel relative to untransfected control cells. Transfection rates were determined as the percentage of positive cells positioned beyond the autofluorescence threshold of the control population.

### SLO penetration assay

This assay has been previously described [16]. Briefly, A549 cells were seeded on Alcian blue-coated glass coverslips in 24-well dishes (40,000 cells/well) and grown for 2 days. Alexa Fluor 488-labeled viruses were bound to cells at 0°C for 60 min. The unbound virus was washed away and the cells were placed into a 37°C water bath for 45 min. After virus internalization, cells were placed on ice, washed twice with streptolysin O (SLO) binding buffer and processed for SLO-mediated perforation of the plasma membrane. Cells were washed twice with SLO binding buffer and incubated with SLO solution (6.2 µg/mL) for 10 min on ice. Unbound SLO was removed by two washes with SLO binding buffer. Cells were then incubated in SLO binding buffer for 5 min at 37°C in a water bath to induce pore formation and immediately returned to ice. After being washed with SLO internalization buffer cells were incubated with anti-Alexa Fluor 488 antibody for 1 h at 0°C. Next, cells were and fixed with 3% paraformaldehyde, permeabilized with 0.5% Triton X-100 in PBS for 5 min and washed once with PBS. Blocking was performed with 10% goat serum in PBS for 5 min at room temperature. Then, cells were incubated with anti-rabbit Alexa Fluor 594 antibody and DAPI for 30 min at room temperature, followed by three 4-min washes in PBS. A post-fixation step was performed with 3% PFA in PBS for 12 min at room temperature. Cells were washed once with PBS, quenched with 25 mM ammonium chloride in PBS for 10 min, washed once more with PBS, and mounted for imaging.

### siRNA experiments

To downregulate specific molecules, we used following predesigned MISSION esiRNA: Rab5 (EHU053901), Rab7 (EHU053221), Rab9 (EMU171281), universal negative control #1 (SIC001), all from Sigma-Aldrich. Cells were transfected at a confluence of 30–50% using Lipofectamine RNAiMax reagent (Invitrogen) according to the manufacturer’s protocol. Final esiRNA concentrations were 10 nM for Rab5 and Rab7, and 5 nM for Rab9. The efficiency of silencing was verified 48 h after transfection by Western blot. Briefly, cells transfected with specific or negative control siRNA were lysed with Laemmli buffer heated to 95°C and sonicated. Proteins were separated by SDS-PAGE and transferred to nitrocellulose membrane. After blocking, the membrane was probed with appropriate primary antibodies, followed by incubation with appropriate horseradish peroxidase-conjugated IgG secondary antibody. Detection was performed with Pierce ECL Western Blotting Substrate using ChemiDoc Imaging System. Densitometry was performed with ImageJ software. Proteins were normalized to the total proteins stained with amido black. The results are presented as the relative expression of proteins compared to cells transfected with control siRNA.

### Transmission electron microscopy

A549 cells grown on glass cover slides were infected with HAdV-D26 or HAdV-C5 at an MOI of 2x10^5^ vp/cell, incubated on ice for 45 min, and then shifted to 37°C for 10 min. After removal of unbound virus particles by washing with fresh medium, cells were incubated for 1 h at 37 °C, after which they were washed twice with PBS and fixed with a mixture of 2% glutaraldehyde and 1% tannic acid in 0.4 M HEPES buffer (pH 7.2). Dehydration was performed using dry ethanol, and heavy metal staining and embedding in EPON resin were carried out on the monolayer according to the protocol described in [46]. Serial sections 70 nm thick were collected from the first 2 µm of the basal cell surface and sections were stained with uranyl acetate and lead citrate for image acquisition, which was performed using a JEOL JEM 1400 transmission electron microscope operated at 120 kV. The number of viral particles present in different cellular compartments was quantified in 56 cells for HAdV-D26 and 42 for HAdV-C5, each one presenting an area greater than 50 µm². The distribution of viral particles among the different compartments was normalized to the total number of quantified particles (262 HAdV-D26 particles, 503 HAdV-C5 particles).

### Confocal microscopy

Cells (2x10^4^ per coverslip) were seeded on coverslips in 24-well plates. Two days later, fluorescently labeled adenoviruses were added to the cells at an MOI 5x10^3^ vp/cell for HAdV-C5 and 10^5^ for HAdV-D26. Because HAdV-26 infects less efficiently A549 cells than HAdV-C5, confocal microscopy was performed under MOIs that yield comparable detectable signals (resulting in up to 50 viruses per cell which allowed for accurate assessment of intracellular localization and trafficking patterns). Cells were than incubated on ice for 45 min to allow viral binding. Subsequently, the cells were transferred to 37°C for the indicated time. The cells were fixed with 2% paraformaldehyde (PFA) in PBS for 12 min at room temperature. Nuclei were labeled with DAPI (4 = ,6-diamidino-2-phenylindole). Coverslips were slide mounted using Fluoromount G (Southern Biotech). Confocal laser scanning microscopy analyses were performed using a Leica TCS SP8 X inverted confocal microscope (Leica Microsystems, Wetzlar, Germany) with 63x/1.40 oil-immersion objective. The images showing intracellular trafficking of Alexa Fluor 488-labeled HAdVs are maximum projections of at least 7 confocal stacks and were processed with the Leica Application Suite X (LAS X) software platform, Adobe Photoshop CC software (Adobe Systems), and ImageJ.

To quantify the intracellular trafficking kinetics of viral particles during the time-course infection (30, 60, 120, 180, and 240 min), a shape-corrected spatial index, termed the Relative Distance Index (Weighted), was calculated for each individual virion (N, number of analyzed virions). First, the absolute Euclidean distances from each viral particle to the nucleus (d1, defined by DAPI staining) and to the plasma membrane (d2, defined by actin staining) were measured in micrometers (µm) using the Euclidean Distance Map (EDM) function in Fiji. To compensate for the characteristic morphological asymmetry of A549 cells and to biologically weigh particles residing in close physical proximity to the nucleus, a non-linear exponential weighting was applied. The Relative Distance Index (Weighted) (I) for each particle was determined using the following formula: I = (d1/ (d1 + d2)) * (1 - EXP(-d1/ 2)). This index strictly constrained the values between 0 (at the nucleus) and 1 (at the plasma membrane). The exponential term served as a mathematical buffer to maintain lower index values for virions within the perinuclear region, preventing local membrane geometry from artificially inflating the distance metrics. For categorical population analysis, virions were assigned to four distinct subcellular fractions based on their absolute distance from the nucleus (d): (i) Nuclear fraction (d = 0 µm), (ii) Perinuclear fraction (0 < d ≤ 3 µm, corresponding to the endoplasmic reticulum and Golgi zones), (iii) Mid-cytoplasmic fraction (3 < d ≤ 7 µm, corresponding to the main cytosol and microtubule transit tracks), and (iv) Cortical/Plasma membrane fraction (d > 7 µm, capturing the cortical actin network and cell periphery). For each time point, the distribution of virions within these four fractions was expressed as a percentage of the total detected viral particles.

### Live cell microscopy

Day before live-cell microscopy, 6x10^4^ U2OS-mCherry-α-tubulin cells were seeded per chamber on 4-chamber glass-bottom dishes (#1.5 glass thickness, iBL). Viral infection was performed on ice with 5x10^4^ MOI and the cells were kept on ice until live cell imaging. The microscopy was performed on Expert Line easy3D STED microscope system (Abberior Instruments) with a 100 x/1.4NA UPLSAPO100x oil objective (Olympus, Tokio, Japan) and avalanche photodiode detector. During imaging, the cells were kept at 37°C and a 5% CO_2_ atmosphere within a heating chamber (Okolab). Images were acquired every ten seconds using Imspector software. Imaging was performed within an hour after infection. Image analysis was performed in Fiji/ImageJ (National Institutes of Health). Quantification and data analysis were performed in GraphPad Prism (GraphPad Software). Tracking of viral particles was done manually by using the Multi-point tool in Fiji. Upon inspection of viral particle movement within the cells, viral particles which could be followed for at least 20 s (three time- frames) were taken into account. Only fast movements towards or from the nucleus were tracked. For evaluation of virus redistribution, virus particles were counted as reaching the nucleus if they were ≤ 1 µm from the nuclear surface.

For co-localization of HAdV-D26 with LysoTracker in live A549 cells, 2x10^4^ cell were seeded on coverslips in 24-well plates. Two days later, fluorescently labeled adenoviruses were added to the cells (MOI 5x10^4^ vp/cell) and incubated on ice for 45 min to allow binding, then 10 min at 37°C, followed by washing with fresh medium to remove unbound viruses, and then incubated at 37°C for the indicated times. In the last 30 min of incubation, LysoTracker Deep Red solution (Invitrogen) was added to the medium to final concentration of 50 nM. After incubation, cells were washed with fresh medium and immediately observed by a Leica TCS SP8 X inverted confocal microscope (Leica Microsystems, Wetzlar, Germany) as stated before. Reflection Interference Contrast Microscopy (RICM) was used to determine cell shape. The co-localization analysis was performed using digital images processed with a co-localization plug-in in Fiji. Manders’ split co-localization coefficient was used to quantify pixel-wise co-localization between HAdV-D26 and LysoTracker signals. Specifically, the coefficient represents the proportion of the HAdV-D26 signal overlapping with the LysoTracker signal, indicating the extent of viral localization within lysosomal compartments. For each time point (120 and 240 min p.i.), a total of 4 independent fields of view containing N = 38 and N = 41 individual cells, respectively, were analyzed. Data are presented as mean ± standard deviation (SD).

### Adenovirus genome delivery to the host nucleus

A549 cells (2x10^4^ per well) were seeded in 6-well plates, and 24 hours after seeding, viral infection was performed on ice with 1x10^4^ MOI in DMEM supplemented with 0.2% (vol/vol) FBS, and incubated on ice for 45 min to synchronize viral entry. After 5 min at 37°C in a humidified incubator with a 5% CO_2_ atmosphere, the medium was removed and replaced with antibiotic-free DMEM supplemented with 10% (vol/vol) FBS. The cells were then kept at 37 °C for defined time points (0, 1, 4, and 8 h).

To monitor viral DNA delivery dynamics in A549 cells after Rab9 silencing, Rab9 knockdown was performed using DharmaFECT 4 on the same day as cell seeding, according to the manufacturer’s protocol. After 48 hours of incubation, viral infection was performed as mentioned above. At the defined time points, cells were washed with PBS, trypsinized and after washing with PBS divided into two fractions. The cell pellet from first fraction was used for total DNA isolation, while the second cell pellet was used for nuclear fraction isolation. For nuclear fraction isolation, 100 µL of hypotonic solution (10 mM HEPES (pH 7.5), 10 mM NaCl, 1 mM DTT, 1 mM EDTA, 2 mM MgCl_2_x6H_2_O) was added to the pellet and incubated for 5 min at room temperature, followed by the addition of 100 µL of 0.05% NP-40 detergent for 10 min. The mixture was vortexed and centrifuged for 2 min at 13,400 × g. The resulting pellet was washed four times in PBS. The final slightly translucent pellet represented the nuclear fraction. Total cellular and viral DNA were extracted using the QIAamp DNA Blood Mini Kit (Qiagen) according to the manufacturer’s instructions. DNA concentration was measured spectrophotometrically at 260 nm, and purity was assessed at 280 nm. The isolated DNA served as a template for qPCR using primers targeting the CMV promoter (forward primer: 5’-TGGGCGGTAGGCGTGTA-’3; reverse primer: 5’-CGATCTGACGGTTCACTAAACG-3’) or the cellular GAPDH gene (forward primer: 5’- AGAACATCATCCCTGCCTCTACTG-3’; reverse primer: 5’ TGTCGCTGTTGAAGTCAGAGGAGA-3’).

### Flow cytometry

Flow cytometry was used to determine the cell surface expression of CAR, CD46 and αvβ3 integrin after transfecting cells with si(Rab9). For analysing cell surface expression of CAR, CD46 and αvβ3 integrin transfected cells were grown in tissue culture dishes, detached with trypsin, and washed twice with PBS. Subsequently, the cells were incubated on ice with the specific primary antibodies that recognized CAR, CD46 and αvβ3 integrin. The binding of unlabelled primary antibodies was revealed by using FITC-conjugated anti-mouse IgG as a secondary reagent. Flow cytometry was done on FACSymphony A1 flow cytometer (BD Biosciences, USA).

### Statistical analysis

All experiments were performed in at least two biological replicas in duplicate or triplicate. All analyses and graphs were created in GraphPad Prism (GraphPad Software Inc., USA). To analyse the intracellular trafficking kinetics of viral particles during the time-course infection, due to the high frequency of absolute zero-values (nuclear fraction) and the inherently skewed, non-normal distribution of the spatial tracking datasets across all time points (N = 261–817 particles per condition), non-parametric methods were implemented. The global effect of infection time on the Relative Distance Index (Weighted) was evaluated using the Kruskal-Wallis test. Upon confirming global statistical significance, Dunn’s multiple comparison post-hoc test with Bonferroni-adjusted p-values was applied to determine specific differences between individual time-point pairs. Other data were analysed by unpaired Student’s t test or Mann-Whitney U test and expressed as mean ± SD or SEM. ns, not significant; *, P < 0.05; **, P < 0.01; ***, P < 0.001; ****, P < 0.0001.

## Supporting information

S1 Fig Intracellular trafficking of HAdV-C5 in epithelial cells. (A) Intracellular trafficking of HAdV-C5 in A549 cells observed by confocal microscopy. Cells were incubated on ice with fluorescently labeled HAdV-C5 (green; MOI 5x10^3^ vp/cell) for 45 min, then 10 min at 37°C, followed by washing with fresh medium to remove unbound viruses. After additional incubation at 37°C for the indicated times, cells were fixed with 2% PFA/PBS and stained with phalloidin-AF555 (red) and DAPI (blue). Representative confocal images are shown (scale bar = 10 μm). The lower panel represents increased selection from the upper panel, with marked edges of the nucleus (blue, based on DAPI) and cell (red, based on phalloidin). (B) Kinetics of HAdV-C5 intracellular trafficking shown in (A). Intracellular localization of individual virions was quantified using the Relative Distance Index (Weighted). An index value of 0 represents particle position at the nucleus, while a value of 1 represents the plasma membrane. Green line represents median. The exponential weighting was applied to compensate for cellular asymmetry. (C) Subcellular distribution of HAdV-C5 shown in (A). Bars represent the percentage of virions (N = 261–444 per time point) localized within four mathematically defined zones based on their absolute distance (d) from the nucleus: nuclear fraction (d = 0 µm), perinuclear region (0 < d ≤ 3 µm), mid-cytoplasm (3 < d ≤ 7 µm) and the cortical periphery (d > 7 µm). (D) HAdV-C5 intracellular localization during the first hour of infection. Representative TEM images of intracellular trafficking of HAdV-C5 in A549 cells. Cells were incubated on ice with HAdV-C5 (MOI 2x10^5^ vp/cell) for 45 min, then 10 min at 37°C, followed by washing with fresh medium to remove unbound viruses, and then incubated for 1 h at 37°C. Cells were then fixed and prepared for TEM. Scale bar = 500 nm. White arrowheads denote virus particles.
(TIF)

S2 Fig Transduction efficiency of HAdV-D26 in A549 and U2OS cell lines. Cells were infected with HAdV-D26 at MOI of 10^4^ vp/cell, and eGFP production was measured at 48 h by flow cytometry. Data are shown as percentage of eGFP positive cells (A) and fold of transduction efficiency (B).
(TIF)

S3 Fig Average velocity of rapid HAdV-C5 movements directed towards or away from the nucleus in U2OS cells. (N = 8 viral particles in both directions).
(TIF)

S4 Fig Co-localization of Alexa Fluor 488-labeled HAdV-C5, HAdV-D26 or HAdV-C2_ts1 with anti-Alexa Fluor 488 antibodies in SLO-treated A549 cells 45 min p.i. Alexa Fluor 488-labeled HAdV-C5, and portion of Alexa Fluor 488-labeled HAdV-D26 particles, but not the penetration deficient Alexa Fluor 488-labeled HAdV-C2_ts1 particles, are accessible to anti-Alexa Fluor 488 antibodies in SLO-treated A549 cells 45 min after internalization. Images shown represent maximum projections of individual confocal stacks. In the merge image, Alexa-488 and Alexa-594 double-positive particles are yellow. Scale bar 10 μm.
(TIF)

S5 Fig Visualization and localization comparison of endogenous, WT, and DN Rab proteins in A549 cells. Panels display characterization of (A) Rab5, (B) Rab7, (C) Rab9, and (D) Rab11. Representative confocal microscopy images across three experimental conditions (rows) are shown: endogenous signal in untransfected cells (top row), or fusion signal in cells transiently transfected with either wild-type (WT) or dominant-negative (DN) versions of each Rab protein (middle and bottom rows, respectively). Each row displays a single field of view imaged through individual channels: nucleus (DAPI, blue), Rab protein, reflection interference contrast microscopy (RICM, gray), and the merged overlay. Individual channels of Rab proteins are presented in grayscale to optimize the visualization of endosomal morphology and signal intensity. In the merged panels, endogenous Rab signals are shown in magenta (detected via specific AF647 antibodies), and transfected WT/DN Rab proteins in red (detected via direct DsRed fluorescence). Scale bar = 25 µm.
(TIF)

S6 Fig Verification of Rab transfection efficiencies by flow cytometry. Representative flow cytometry histograms displaying the expression of DsRed-tagged wild-type (WT) and dominant-negative (DN) constructs for Rab5, Rab7, Rab9, and Rab11 in A549 cells. In each panel, three overlapping histograms are presented: untransfected control cells (ctrl; gray), cells transfected with the WT Rab construct (blue), and cells transfected with the DN Rab construct (orange). The transfection efficiency for each construct is indicated adjacent to the respective histogram and is expressed as the percentage of DsRed-positive cells determined relative to the untransfected baseline control population.
(TIF)

S7 Fig Expression of Rab5, Rab7 or Rab9 proteins in A549 cells transfected with non-specific or Rab specific siRNA. The efficiency of Rab5, Rab7 or Rab9 protein downregulation was evaluated by SDS-PAGE protein separation and western blotting using Rab specific antibodies (Ab) 48 h after transfection. The numerical values shown in the figure represent the normalized ratio of the chemiluminescent signal of the Rab5-, Rab7- or Rab9-specific bands to the total protein signal (amido black staining) relative to cells transfected with a non-specific siRNA (si(-)).
(TIF)

S8 Fig Cell surface expression of αvβ3 integrin, CAR and CD46 on A549 cells after downregulating Rab9. A549 cells were transfected with 5 nM si(-) or si(Rab9) and 48 h after cells were detached, incubated with specific antibodies on ice, and cell surface expression of αvβ3 integrin, CAR and CD46 was analyzed by flow cytometry.
(TIF)

S1 Raw Gel Original uncropped and unadjusted images underlying Western blot results presented in this manuscript. (PDF)
