# Supplementary material for: Rab9 depletion enhances human adenovirus type 26 transduction efficiency through increased internalization and reduced late endosomal/lysosomal retention
Source: PLoS Pathog. 2026 Aug 10;22(8):e1014028. doi: 10.1371/journal.ppat.1014028 (PMC13480637; doi:10.1371/journal.ppat.1014028)

Rab5

Sample 1 (S1) – A549 transfected with si(-)
Sample 2 (S2) – A549 transfected with si(Rab5)

Membrane – amido black (original color)

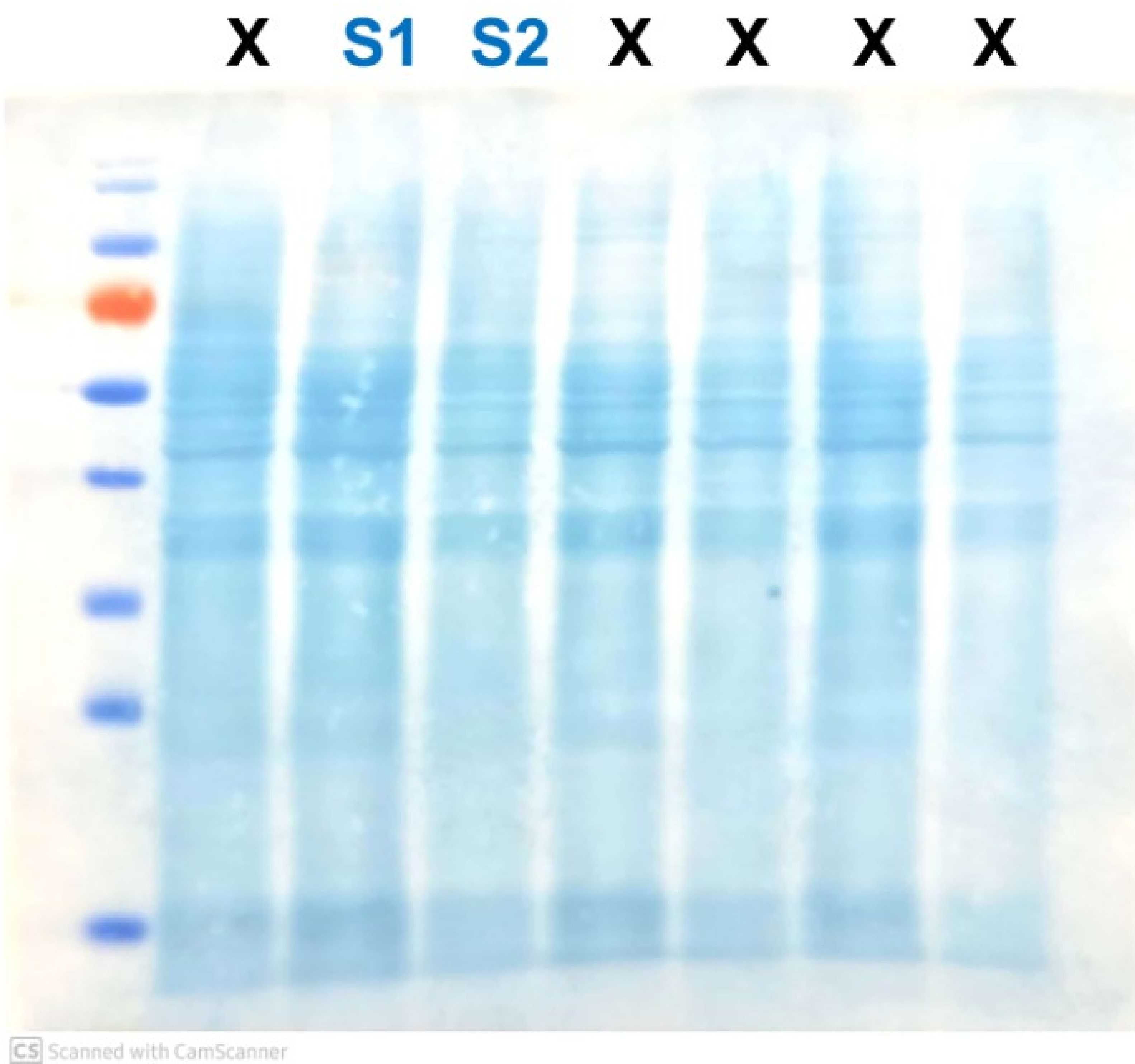

Membrane (amido black):
Cropped region (purple box) converted to grayscale for final figure preparation.

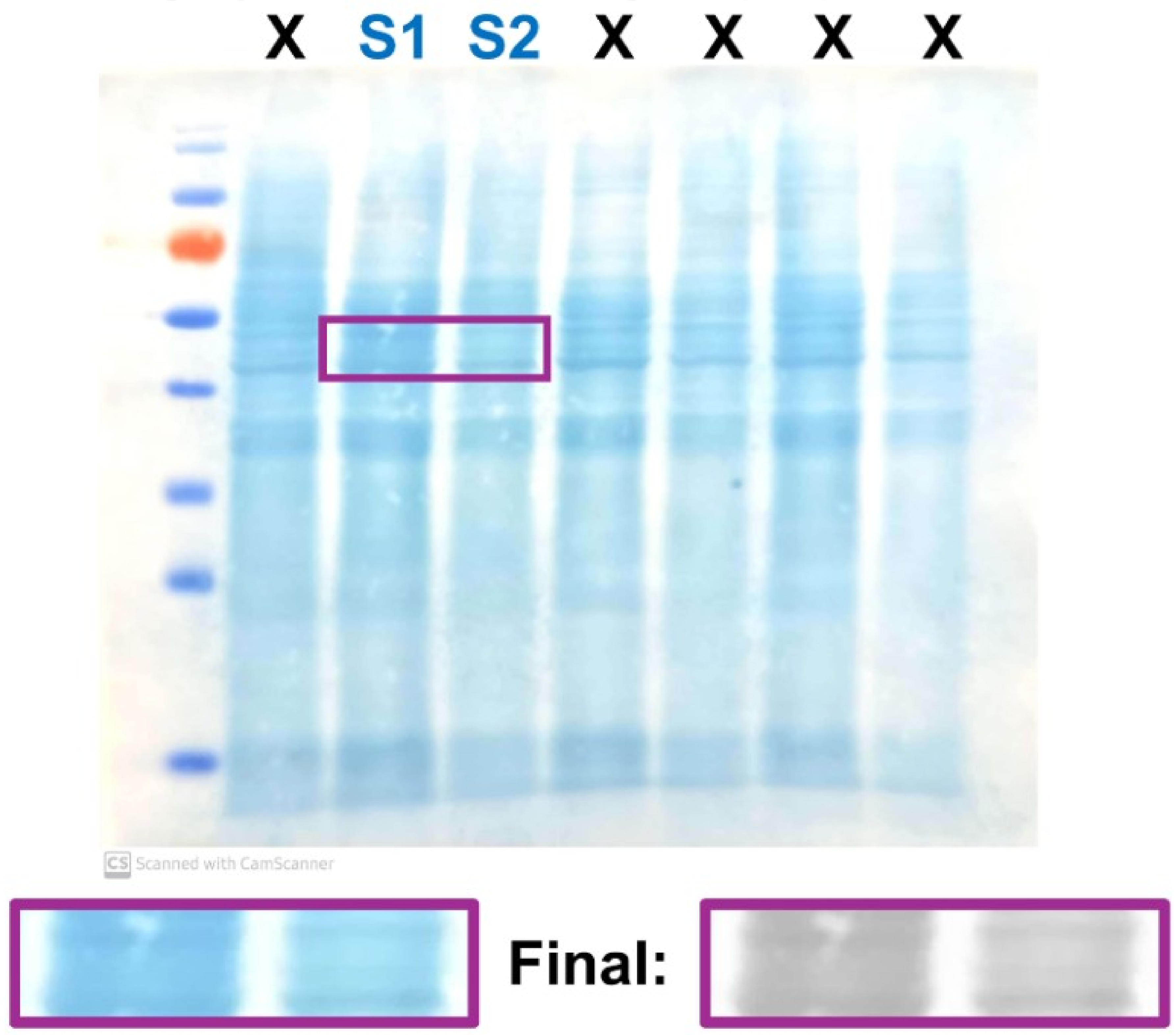

Membrane (anti-Rab5 with marker overlay):
Cropped region (purple box) showing the anti-Rab5 signal with the 25 kDa marker indicating relative position.

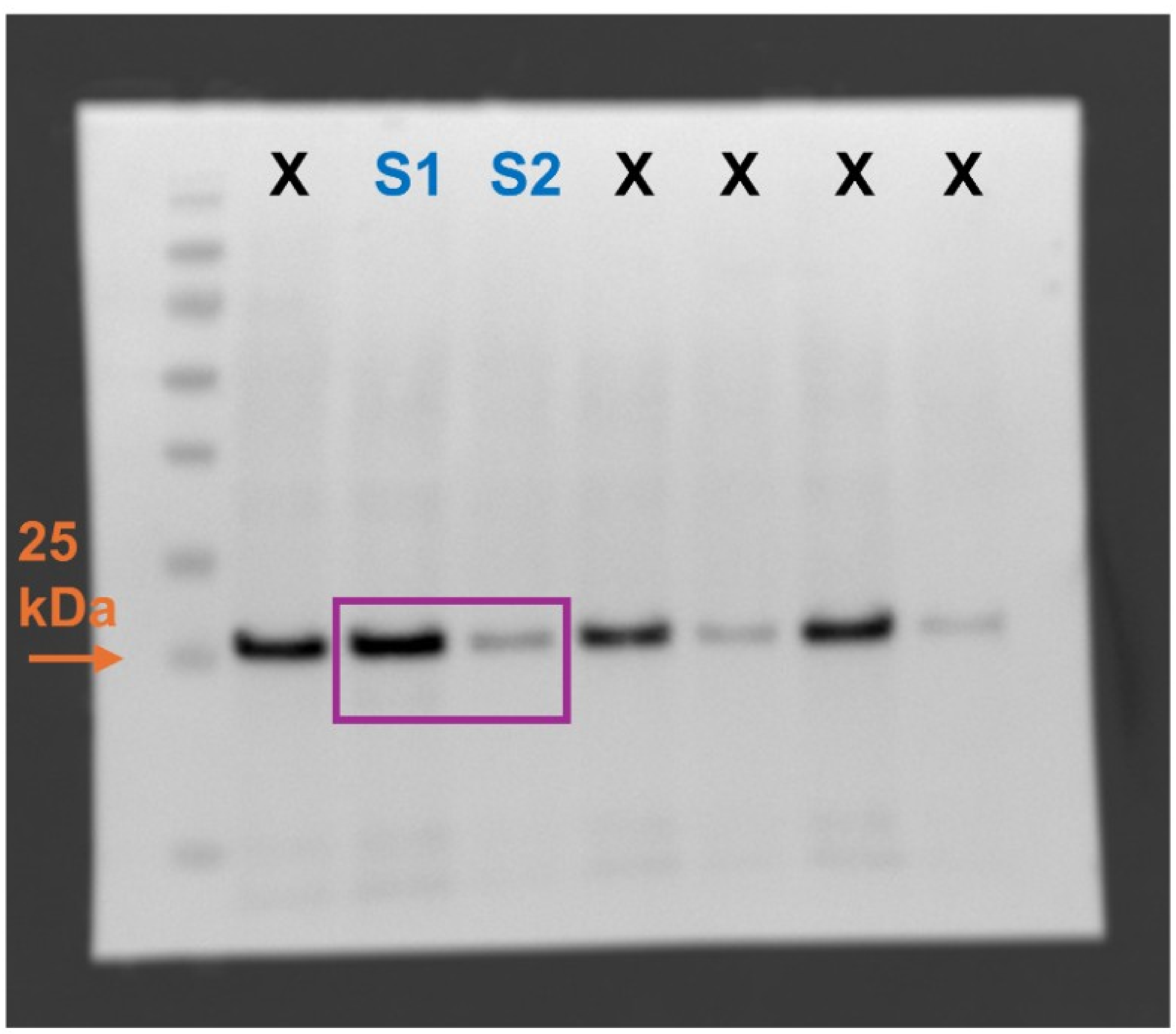

Membrane – anti-Rab5 (original photo with labelled samples)

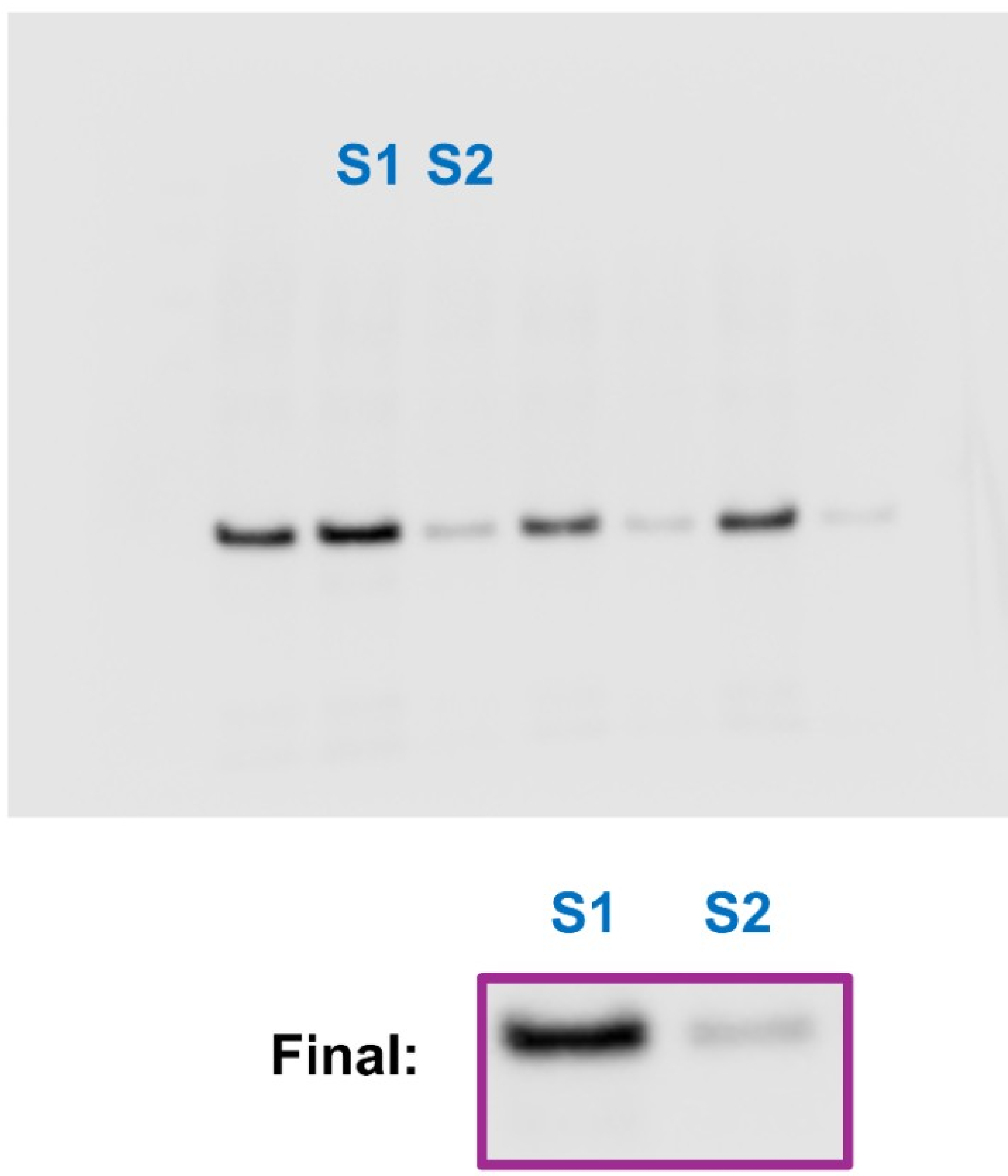

Rab7

Sample 1 (S1) – A549 transfected with si(-)
Sample 2 (S2) – A549 transfected with si(Rab7)

Membrane – amido black (original color)

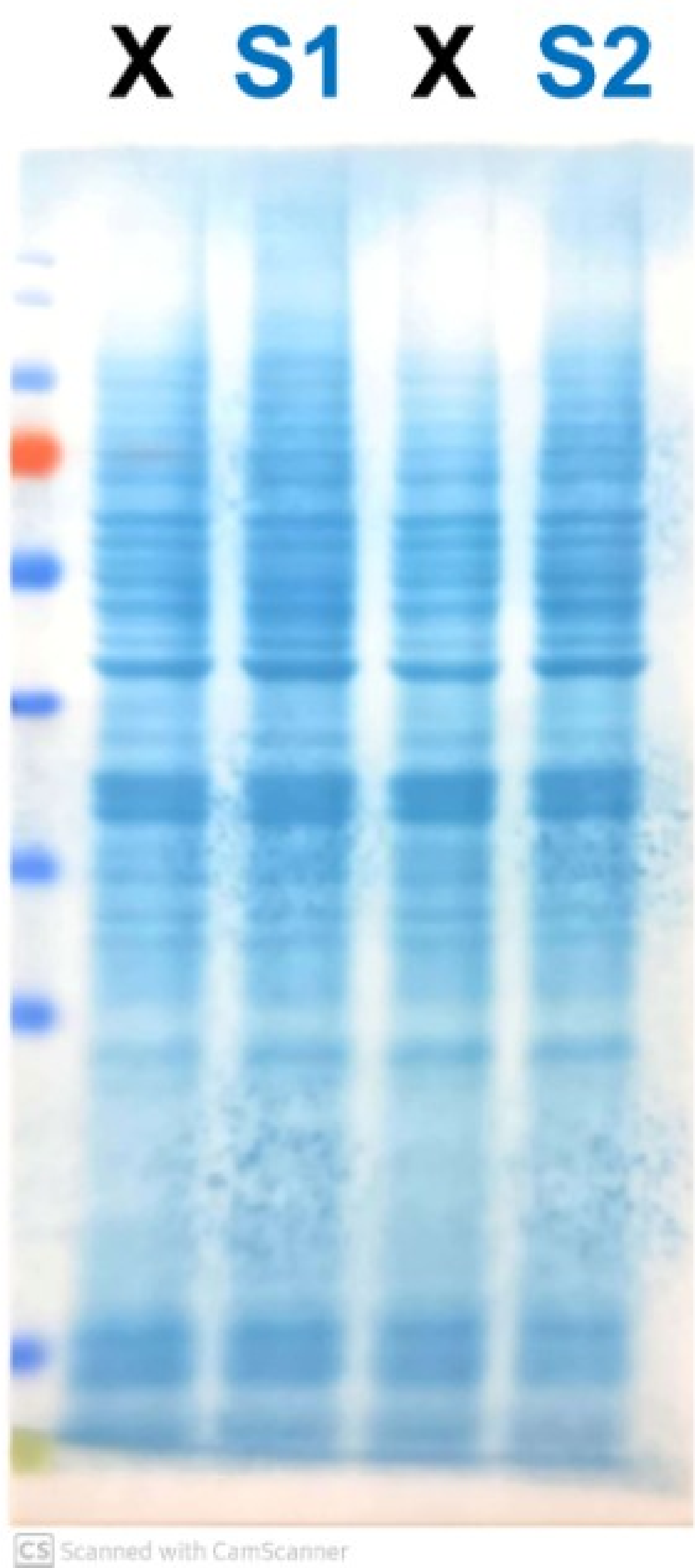

Membrane (amido black):
Cropped region (purple box) converted to grayscale for final figure preparation.

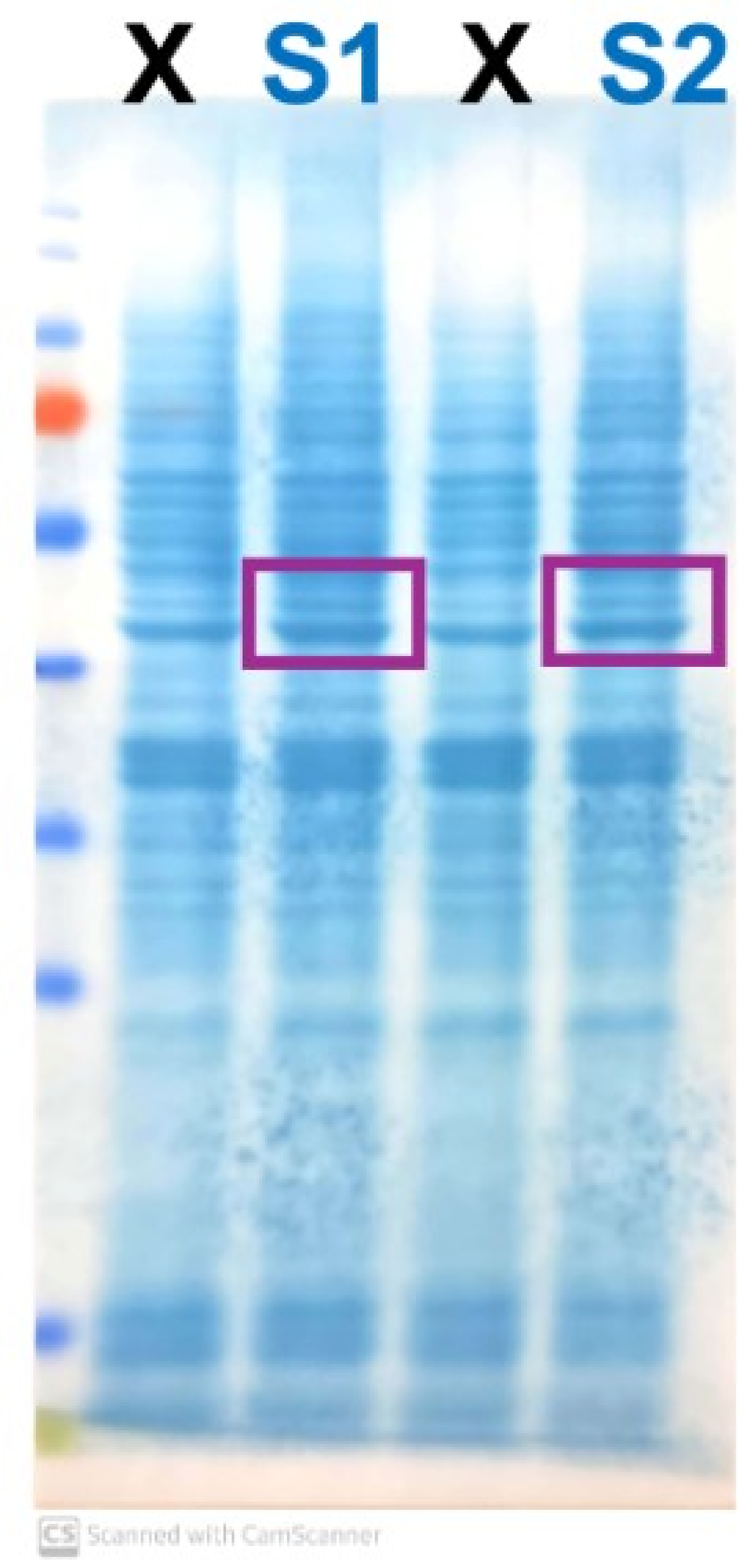

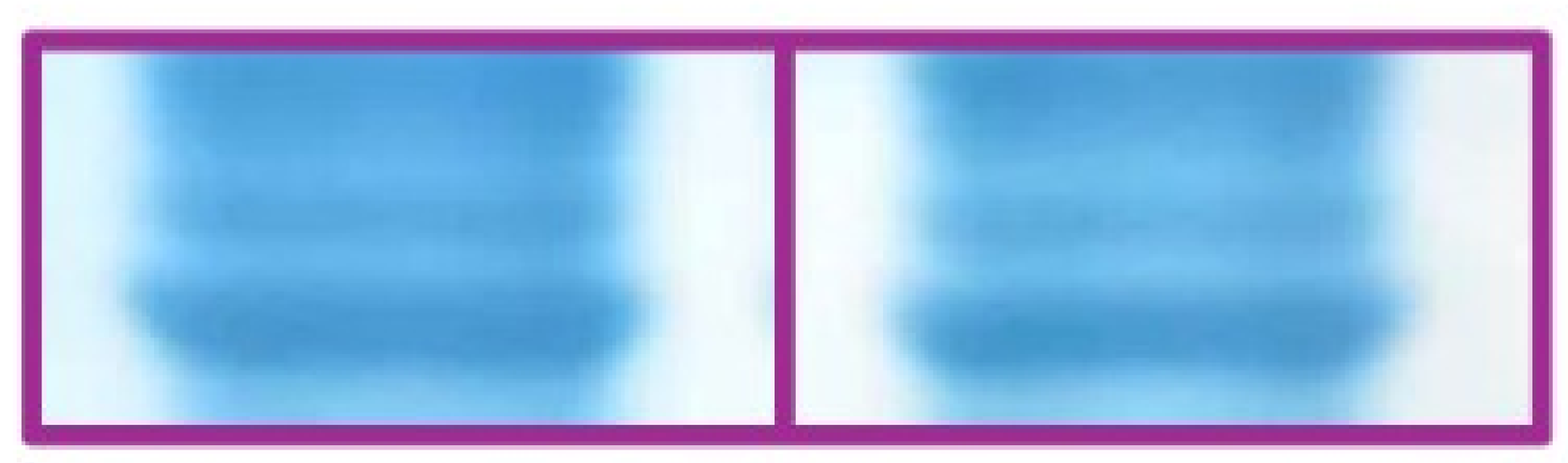

Final:

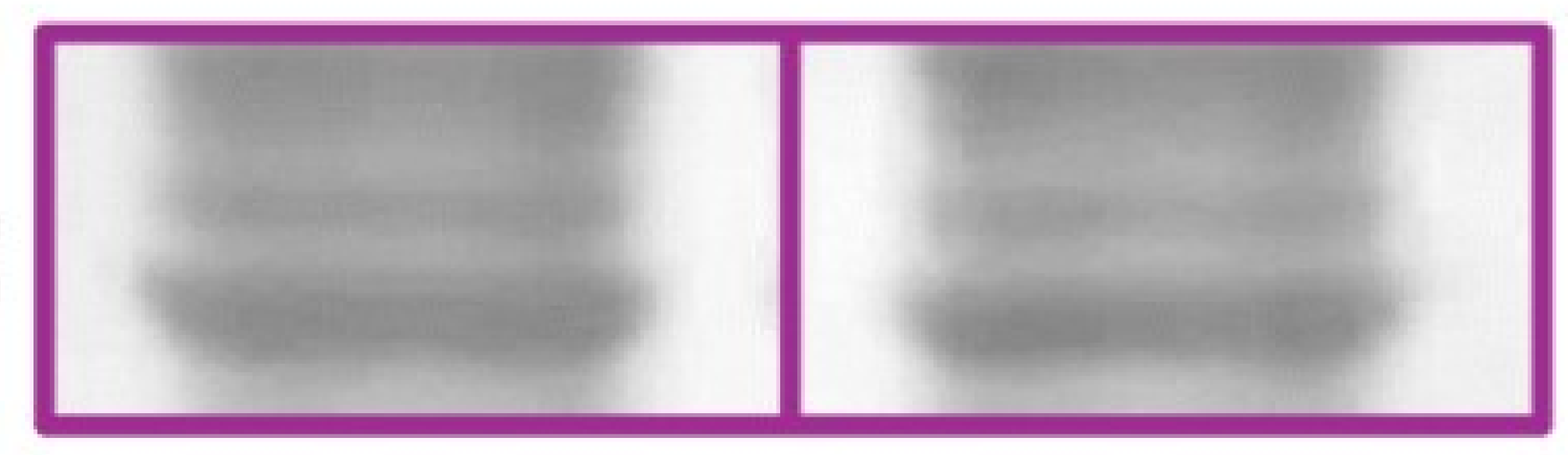

Membrane (anti-Rab7 with marker overlay):
Cropped region (purple box) showing the anti-Rab7 signal with the 25 kDa marker indicating relative position.

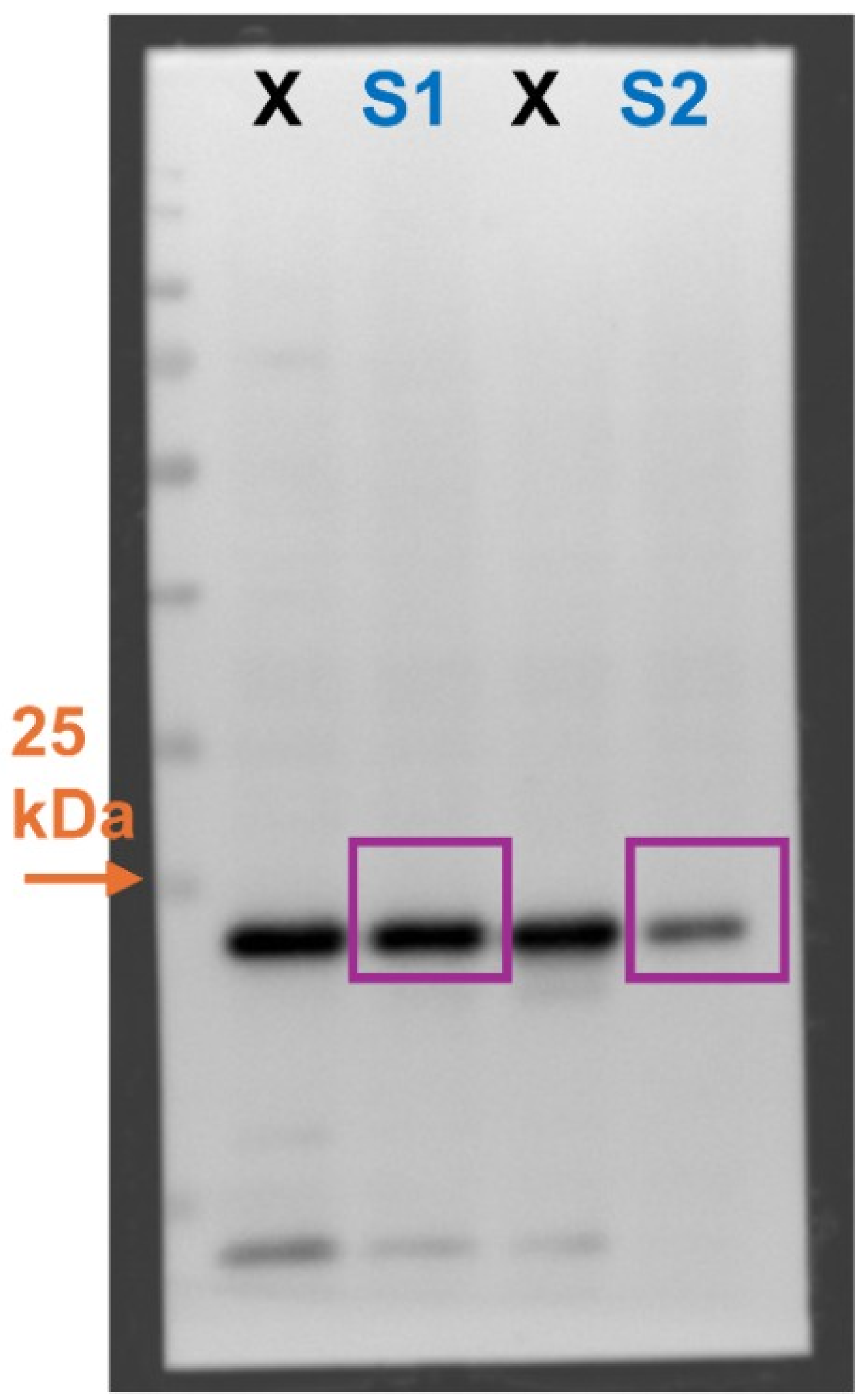

Membrane – anti-Rab7 (original photo with labelled samples)

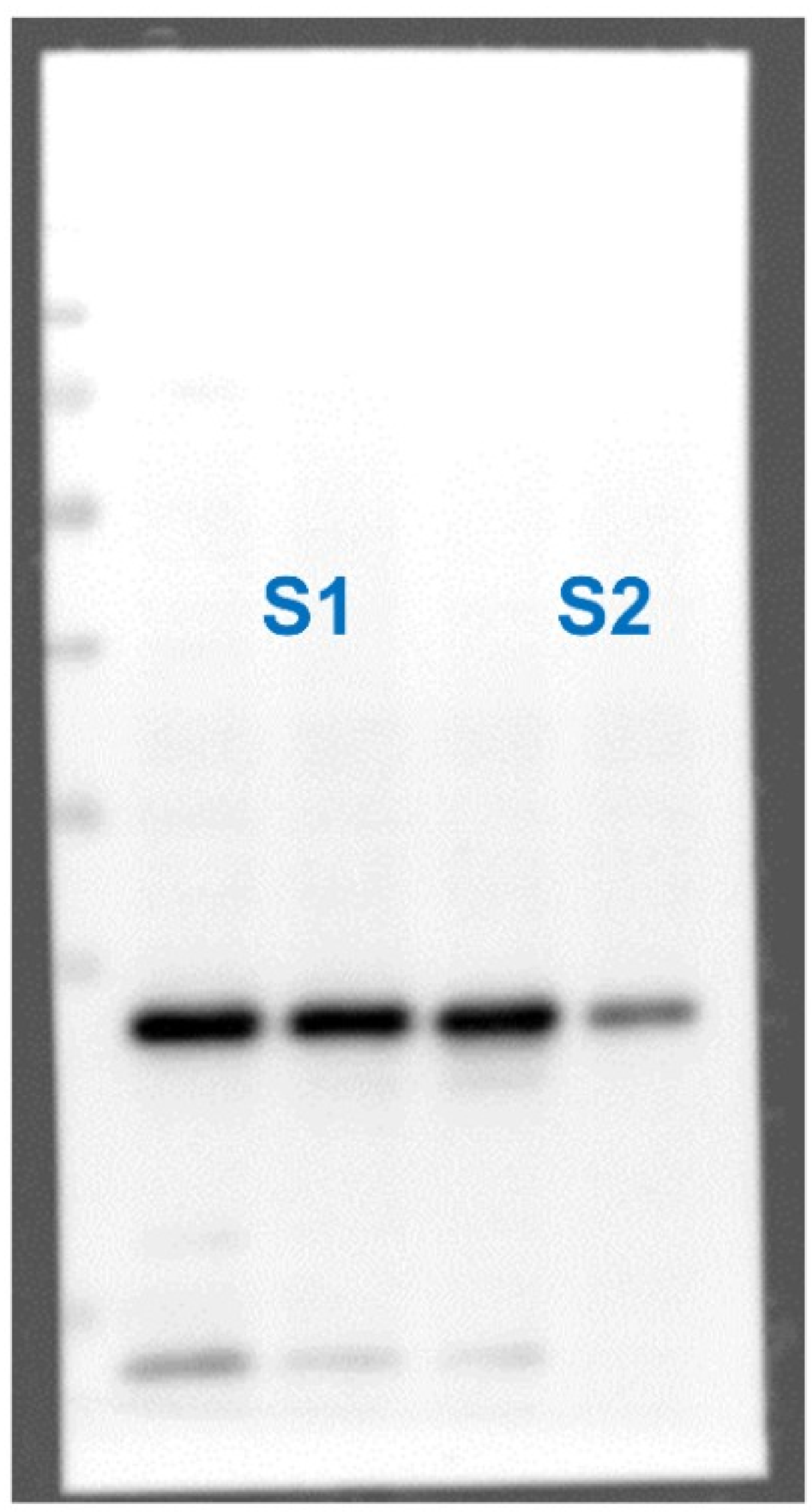

Final:

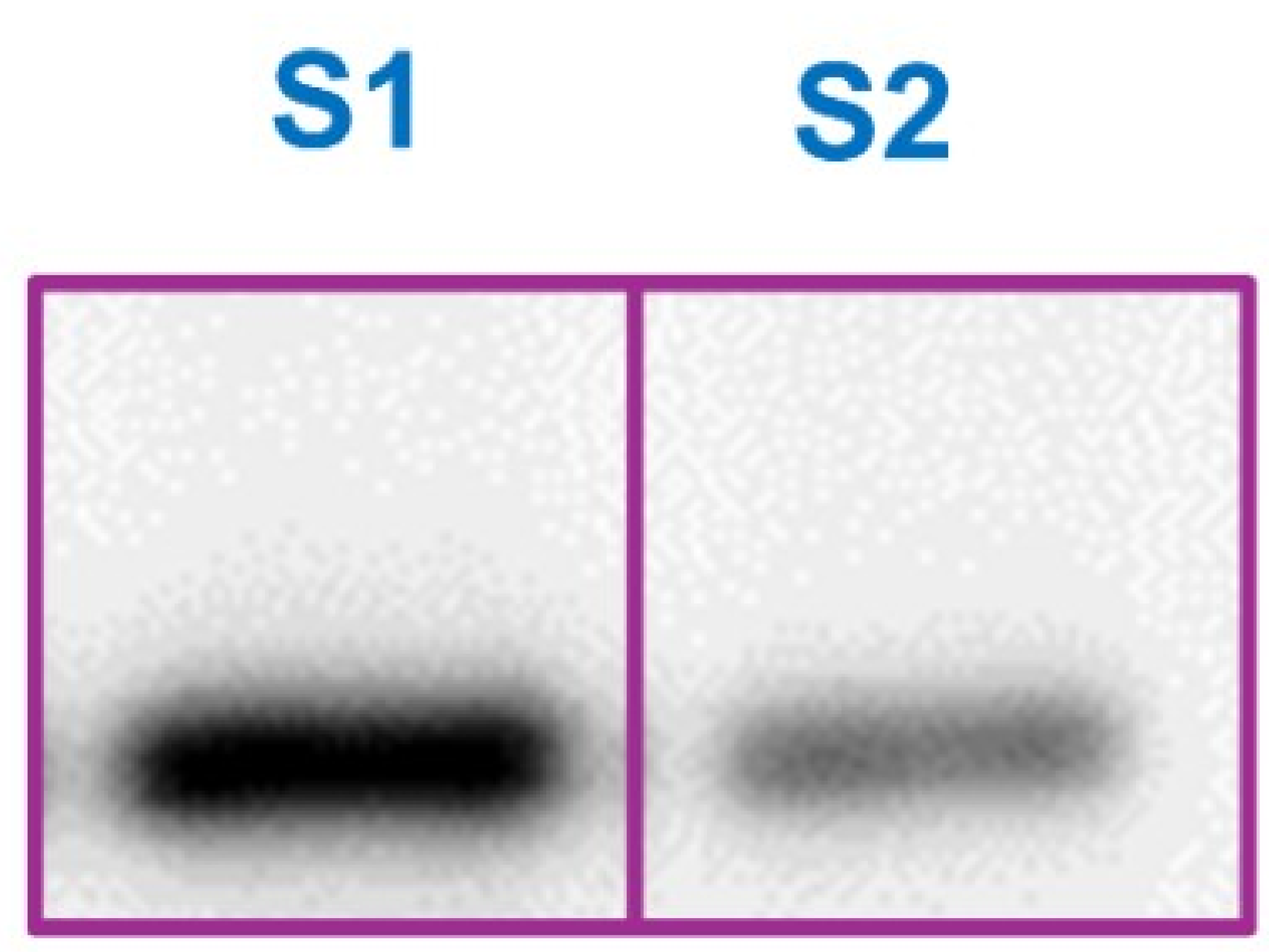

Rab9

Sample 1 (S1) – A549 transfected with si(-)
Sample 2 (S2) – A549 transfected with si(Rab9)

Membrane – amido black (original color)

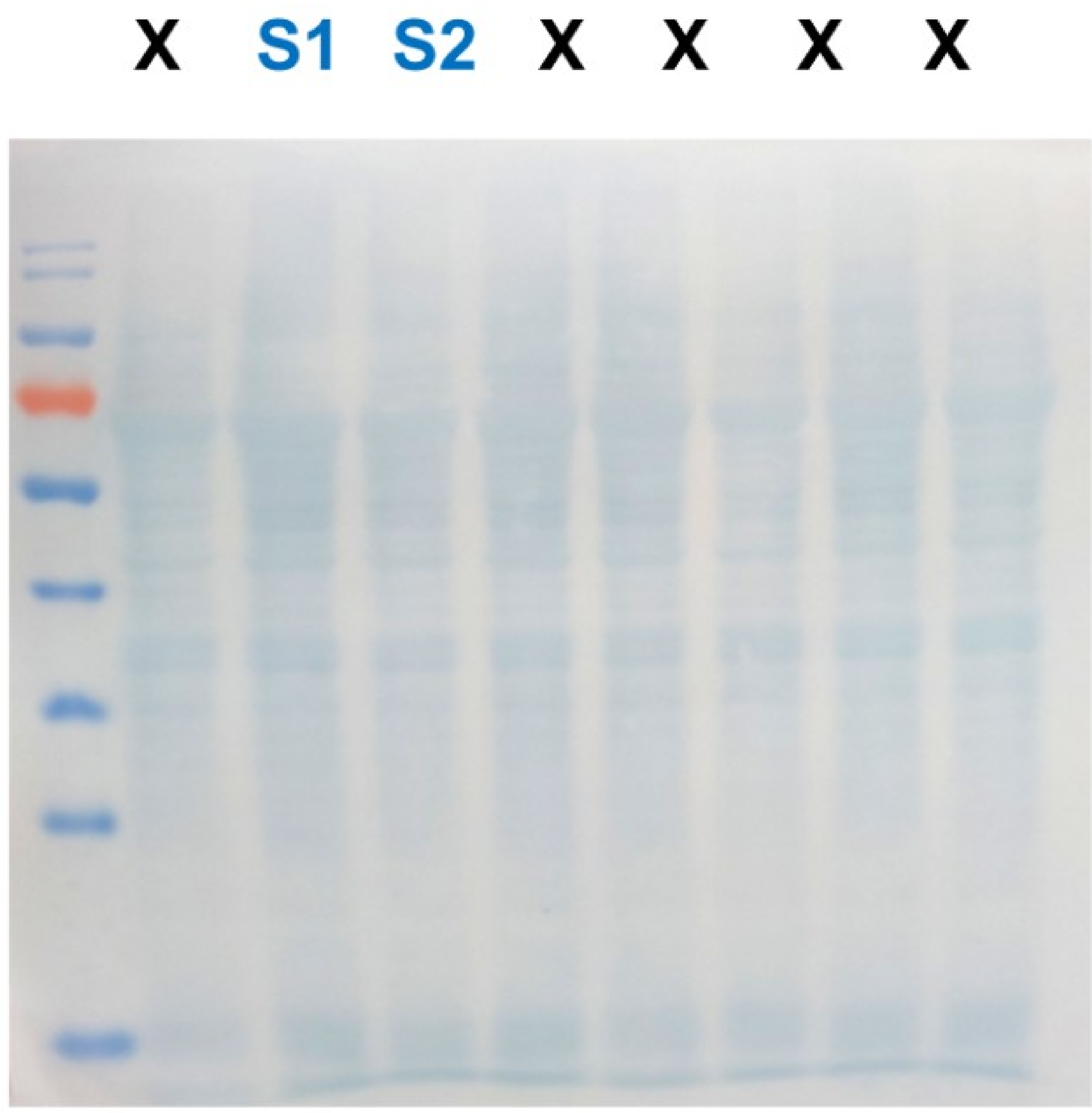

Membrane (amido black):
Cropped region (purple box) converted to grayscale for final figure preparation.

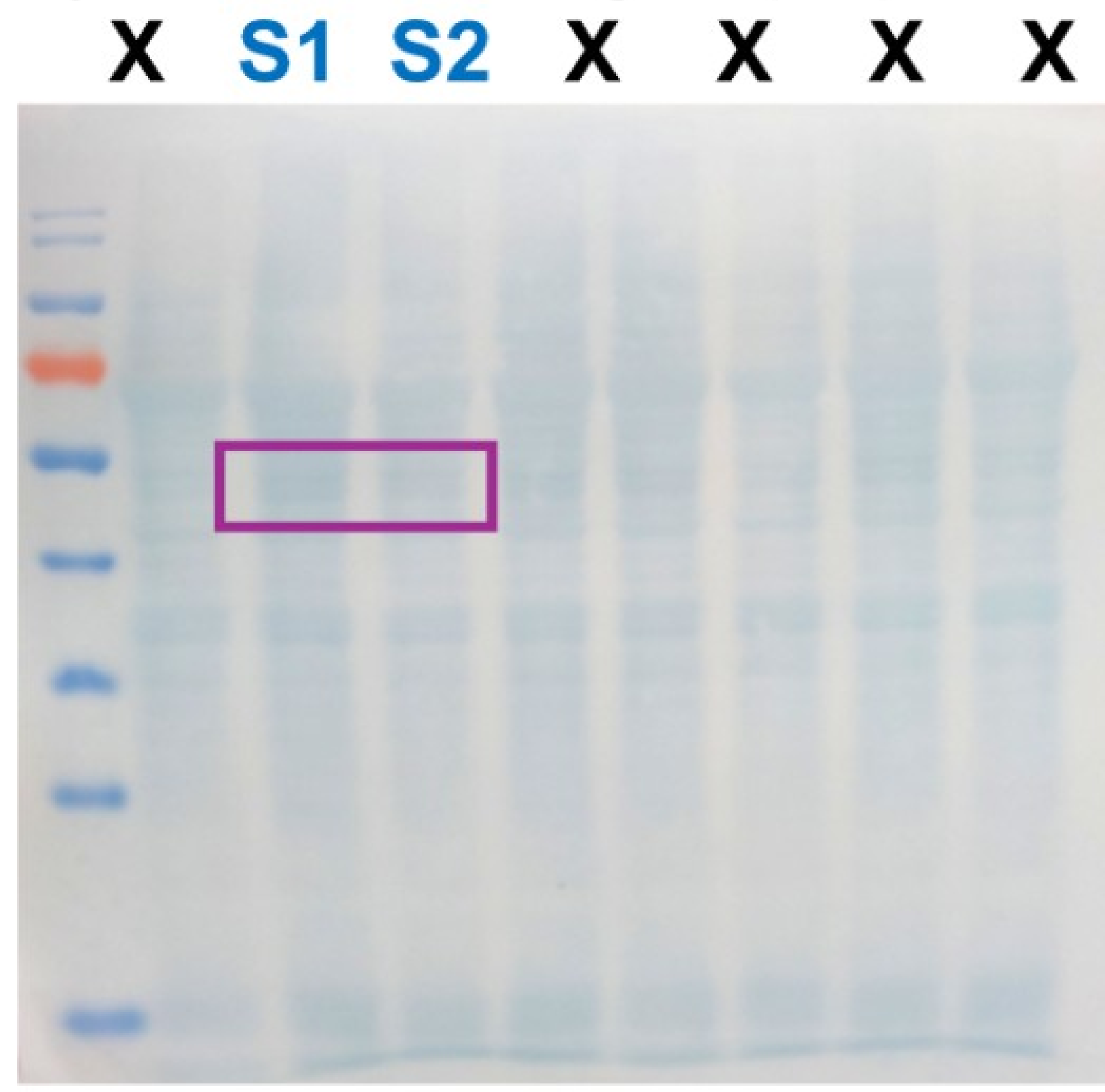

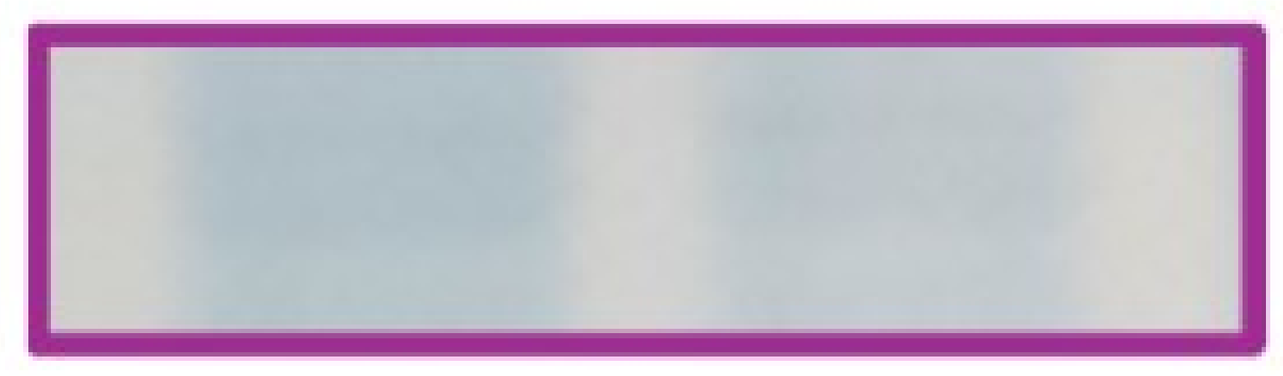

Final:

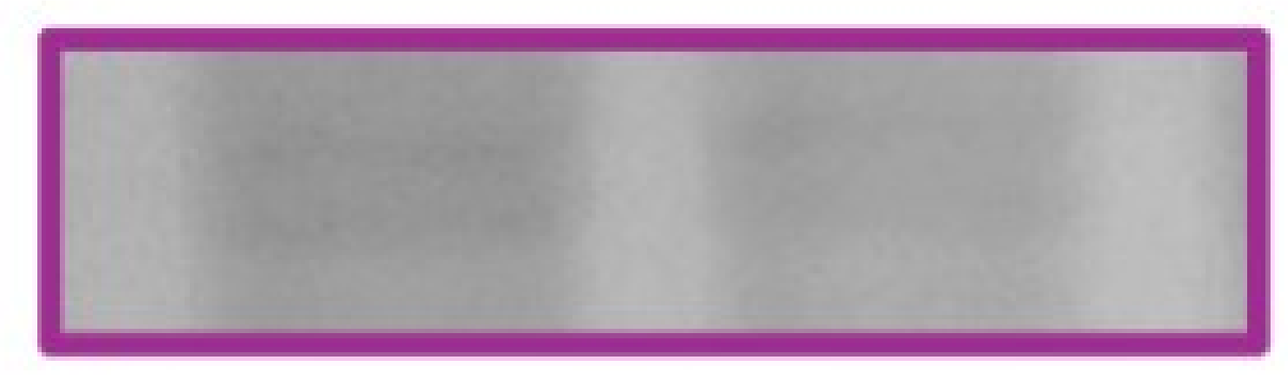

Membrane (anti-Rab9 with marker overlay):
Cropped region (purple box) showing the anti-Rab9 signal with the 25 kDa marker indicating relative position.

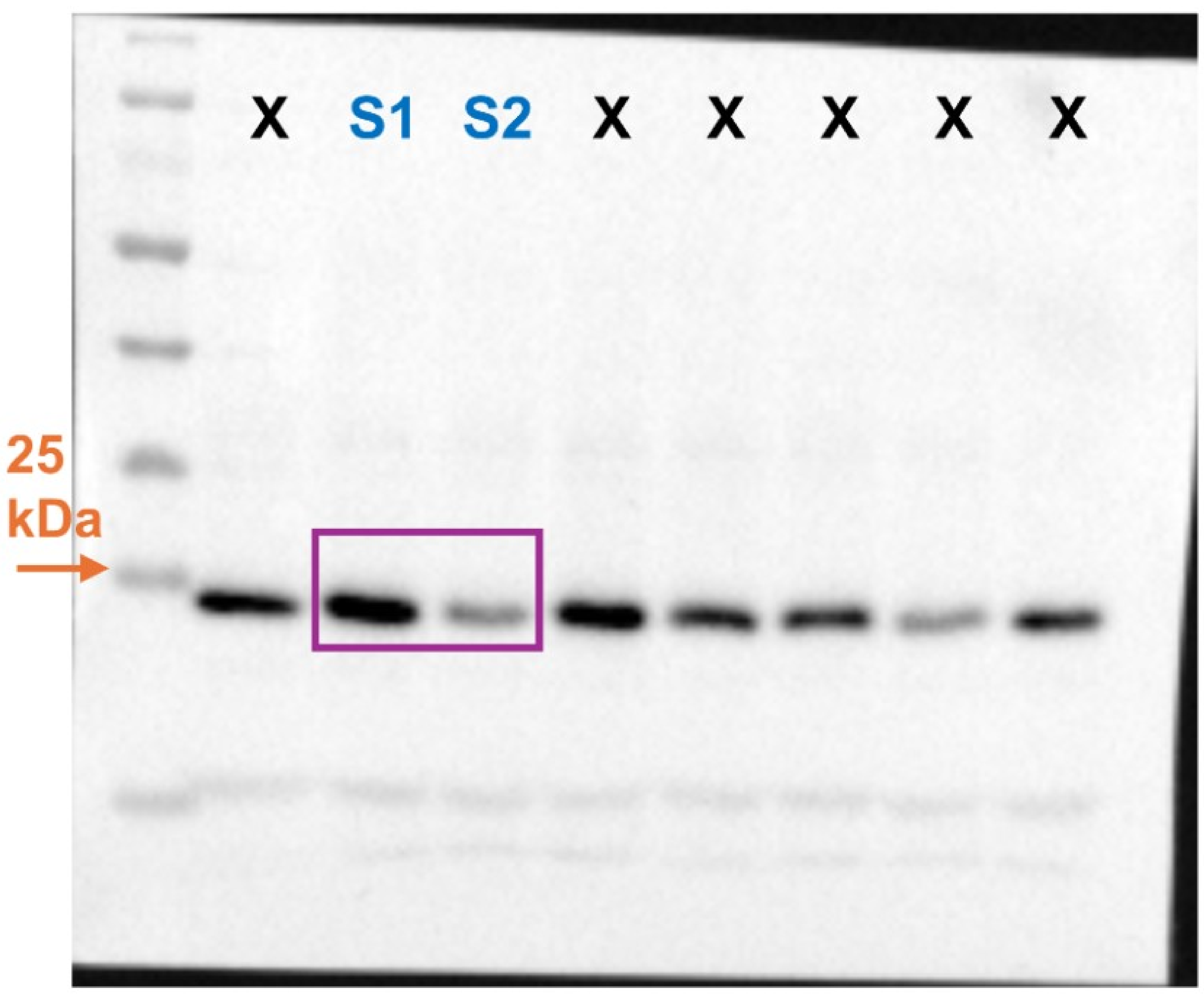

Membrane – anti-Rab9 (original photo with labelled samples)

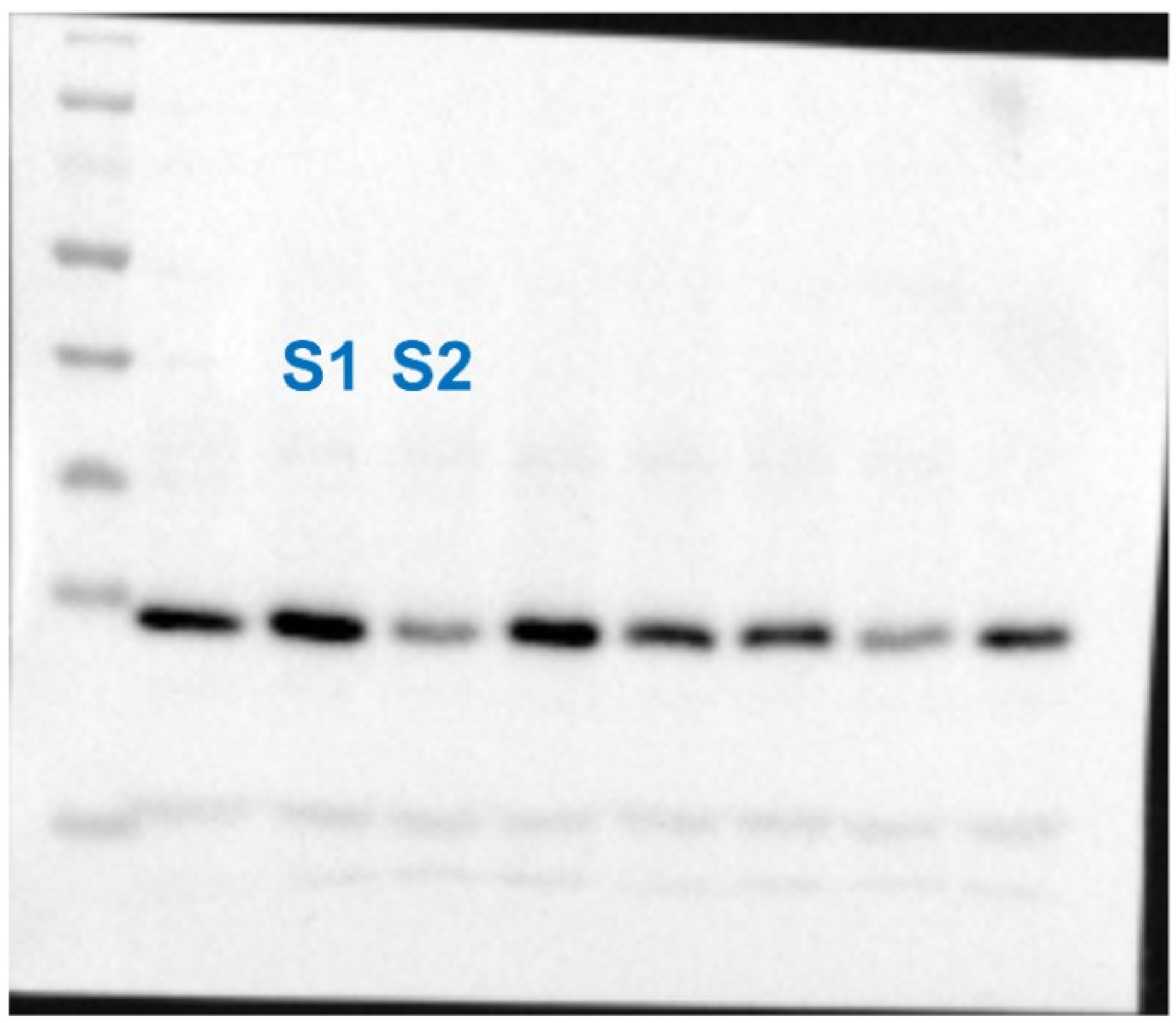

Final:

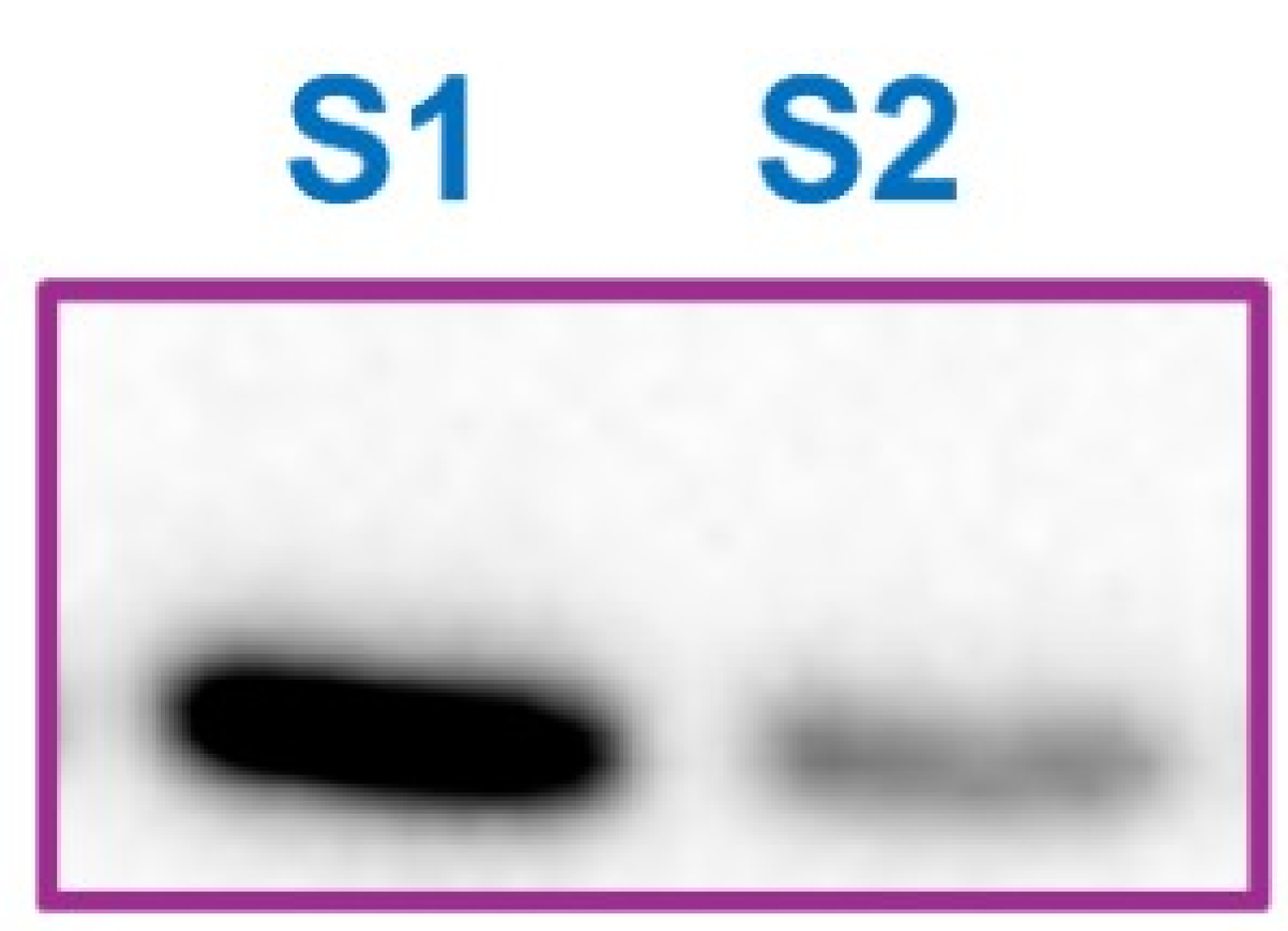

Supplement: Supplementary file 1 [file ppat.1014028.s009.pdf]
